# Optimising the method to develop spheroids from MDA-MB-468 human triple negative breast cancer cells

**DOI:** 10.1007/s11033-026-11451-4

**Published:** 2026-01-24

**Authors:** Shaza M. Mohamedahmed, Muhamad Noor Alfarizal Kamarudin, Premdass Ramdas, Usha Sundralingam, Ammu K. Radhakrishnan

**Affiliations:** 1 Jeffrey Cheah School of Medicine and Health Sciences, Monash University Malaysia, Bandar Sunway, Sunway, 475000 Selangor Malaysia; 2 School of Pharmacy, Monash University Malaysia, Sunway, 47500 Selangor Malaysia

**Keywords:** Triple-negative breast cancer, Spheroids, Gene markers

## Abstract

**Purpose:**

Three-dimensional (3D) spheroid models are increasingly used to emulate the tumour microenvironment for preclinical drug screening. This study aimed to optimise and assess spheroid formation from MDA-MB-468 triple-negative breast cancer (TNBC) cells using hanging drop, liquid overlay, and rigid scaffold methods under normal oxygen (NOC) and low oxygen (LOC) culture conditions.

**Methods:**

Spheroids were generated and characterised using bright-field microscopy with AnaSP morphometrics (sphericity, solidity, and perimeter). Gene expression of Epithelial-Mesenchymal Transition (EMT), stemness, and hypoxia/angiogenesis markers (CD44, HIF1A, VEGFA, TWIST1, SNAI1, and NES) was quantified using qPCR. The optimised model was further evaluated using field-emission scanning electron microscopy (FE-SEM) and Hoechst fluorescence.

**Results:**

A workflow combining hanging-drop pre-aggregation with ultra-low attachment (ULA) or agarose-coated plates under NOC produced consistent, compact spheroids. Scaffold cultures formed rapidly but showed size variability under NOC and LOC. Across methods, spheroids were less compact, and gene expression patterns deviated from expected hypoxic responses. ***HIF1A*** and ***VEGFA*** were more highly expressed under NOC, suggesting pseudo-hypoxic signalling and activation of angiogenesis-related pathways. ***CD44*** and ***TWIST1*** were upregulated in most spheroid types, whereas ***SNAI1*** and ***NES*** exhibited condition- and method-specific variability.

**Conclusion:**

Spheroids cultivated under normoxic conditions demonstrated enhanced structural integrity and transcriptional fidelity. Nonetheless, the study identified that the most compact and resilient spheroids were achieved through the use of hanging-drop pre-aggregation combined with ULA-plates under NOC. The enhanced structural integrity and transcriptional fidelity observed in these spheroids make them valuable models for studying cancer biology and drug responses.

**Supplementary Information:**

The online version contains supplementary material available at 10.1007/s11033-026-11451-4.

## Introduction

Preclinical evaluation of cancer therapeutic agents typically progresses from in vitro studies to animal models before advancing to clinical trials [1, 2]. Therefore, cancer cell lines play a crucial role in understanding cancer biology, developing novel therapeutic and diagnostic strategies [3], as well as offer accessible andreproducible models for evaluating drug efficacy to elucidate possible molecular mechanisms [4]. Using cell lines in cancer research to identify new therapeutic agents is challenging because of the low proportion of samples yielded [5, 6]. Additionally, most human cancer cell lines typically originate from a limited number of patient samples. They may not encompass all cancer types and ethnic groups [5, 6]. Traditional two-dimensional (2D)/monolayer culture models lack the structural and physiological complexity of in vivo tumours, limiting their predictive value [7, 8].

Three-dimensional (3D) culture systems have emerged as more physiologically relevant models that mimic essential aspects of the tumour microenvironment, including cell-cell and cell-matrix interactions, drug penetration barriers, and hypoxia [9, 10]. Various 3D culture systems have been developed, each offering specific advantages. Simple methods such as hanging drop and low-adhesion plates enable spheroid formation without external scaffolds. At the same time, more complex systems may incorporate hydrogels or extracellular matrix components to support 3D culture development [11, 12]. However, replicating tissue-tissue interfaces, accurately modelling mechanical microenvironments, and diverse oxygen and nutrient gradients remain challenging [13]. 3D culture (cell lines) generates cellular aggregates that are broadly classified according to their level of structural organisation and intercellular cohesion [14]. These include loose aggregates, compact aggregates, and fully developed spheroids. Loose aggregates are early formations composed of loosely associated cells with irregular borders and weak adhesion. Compact aggregates demonstrate improved cohesion and a more uniform, rounded morphology and exhibit apparent surface irregularities. Fully developed spheroids display a well-defined contour and stratified architecture, typically comprising an outer proliferative layer, an intermediate quiescent zone, and an inner hypoxic or necrotic core. The progression from loose to compact to mature spheroids reflects increasing cellular compaction, enhanced extracellular matrix (ECM) deposition, and improved physiological relevance, thereby mimicking some of the essential features of in vivo tumour microenvironments [14].

Key variables such as cell density, matrix composition, and oxygen availability significantly influence spheroid formation, structure, and functionality [15, 16]. Spheroids’ size and structural heterogeneity introduce additional variability that affects reproducibility and drug testing outcomes [17, 18]. Furthermore, gene expression analysis within 3D models can offer valuable insights into tumour progression, therapeutic resistance, and metastatic potential [19]. Notably, markers such as cluster of differentiation 44 (CD44) [20, 21], hypoxia-inducible factor-1alpha (*HIF1A*) [22], snail family transcriptional repressor 1 (*SNAI1*) [23] and twist basic helix-loop-helix transcription factor 1 (*TWIST1*) are frequently investigated in the context of epithelial-to-mesenchymal transition (EMT), hypoxia adaptation, and stemness [24].

Breast cancer (BC) continues to be the most frequently diagnosed malignancy among women globally, characterised by significant heterogeneity [25]. Among its subtypes, triple-negative breast cancer (TNBC), distinguished by the absence of oestrogen receptor (ER), progesterone receptor (PR), and human epidermal growth factor receptor 2 (HER2) expression [26], and is associated with aggressive clinical behaviour, limited treatment options, and poor prognosis. TNBC cell lines, namely MDA-MB-468 [27], are commonly used for preclinical studies [28, 29]. Still, they pose specific challenges in spheroid culture due to their poor self-aggregation capacity, high maintenance requirements, and the need for specialised tools and reagents to generate biologically relevant 3D structures [30]. These factors collectively make the process technically challenging and financially burdensome, especially compared to more spheroid-prone lines such as hormone receptor-positive MCF-7 [31].

This study aimed to define a robust 3D workflow for MDA-MB-468 cells. We systematically compared hanging-drop pre-aggregation, collagen-supplemented liquid overlay, and rigid scaffold systems under 19% vs. 4% O₂, prioritising compactness/structural integrity and restricting gene expression profiling to models that achieved relatively compact spheroids. The model meeting these criteria was further evaluated using Field Emission Scanning Electron Microscopy (FE-SEM) and fluorescence microscopy.

## Methods

### Cell line

Roswell Park Memorial Institute (RPMI) (GIBCO, Cat. No.12633012, USA), foetal bovine serum [FBS] (GIBCO, Cat. No.16000044, USA), N-2-hydroxyethylpiperazine-N-2-ethane sulfonic acid penicillin-streptomycin solution (P/S) (10,000 U/mL) (GIBCO, Cat. No. 15140122, USA), phosphate-buffered saline [PBS] pH7.2 (GIBCO, Cat. No. 20012027, USA); TrypLETM express enzyme (1X) (GIBCO, Cat. No. 12604021, USA); agarose, high EEO, for molecular biology (Sigma-Aldrich, CAS Number: 9012-36-6, USA). The human TNBC cell line, MDA-MB-468MDA-MB-468 (ATCC^®^ HTB-132™); MDA-MB-231 (ATCC^®^ HTB-26™).

#### Two-dimensional cell culture Preparation

MDA-MB-468 human TNBC cells (ATCC HTB-132) were cultured in complete medium, which consisted of RPMI supplemented with 10% FBS and 1% P/S. The cells were sub-cultured as recommended by ATCC. The cell count was adjusted from (1–10) × 10^3 cells/20 µL using complete medium.

#### Spheroid culture development

##### Hanging drop (HD)

**Fig. 1:**
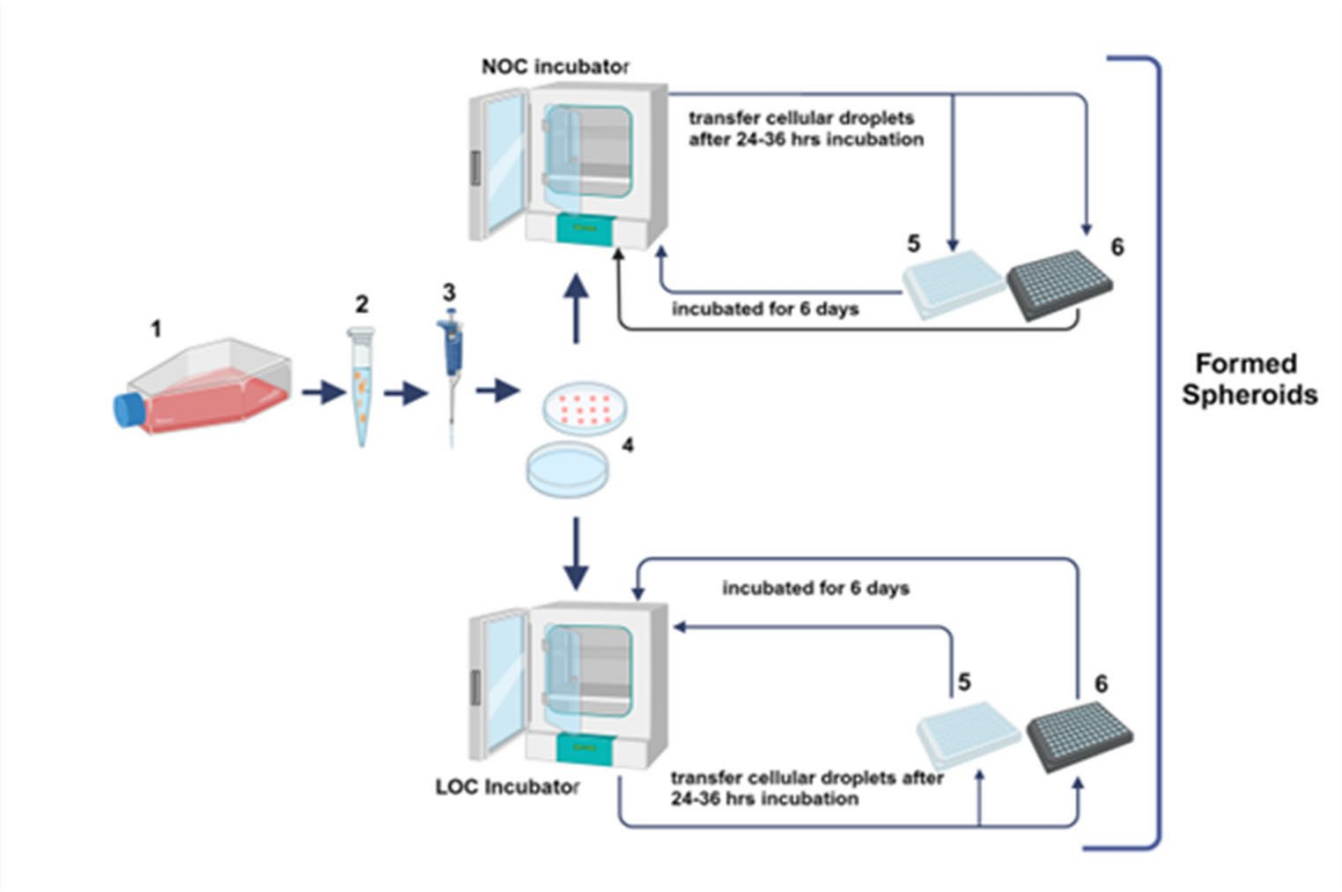
The illustration shows the process of forming the spheroid culture from the two-dimension culture [1]single**-** cell suspension(cell density 5 × 10^3 cells/20 µL) [2], using 20 µl micropipettes [3], and the preparation of hanging drop culture [4], which is incubated under LOC and NOC for 24–36 hs, then transferred to agarose coated-96well culture plate [5] or ULA-plates [6] under LOC or NOC. [**created with bioRender**,** https://www.biorender.com]. NOC: normal oxygen condition; LOC: Low oxygen condition; ULA-plates: ultra-low attachment plate; hs: hours**

**Fig. 2:**
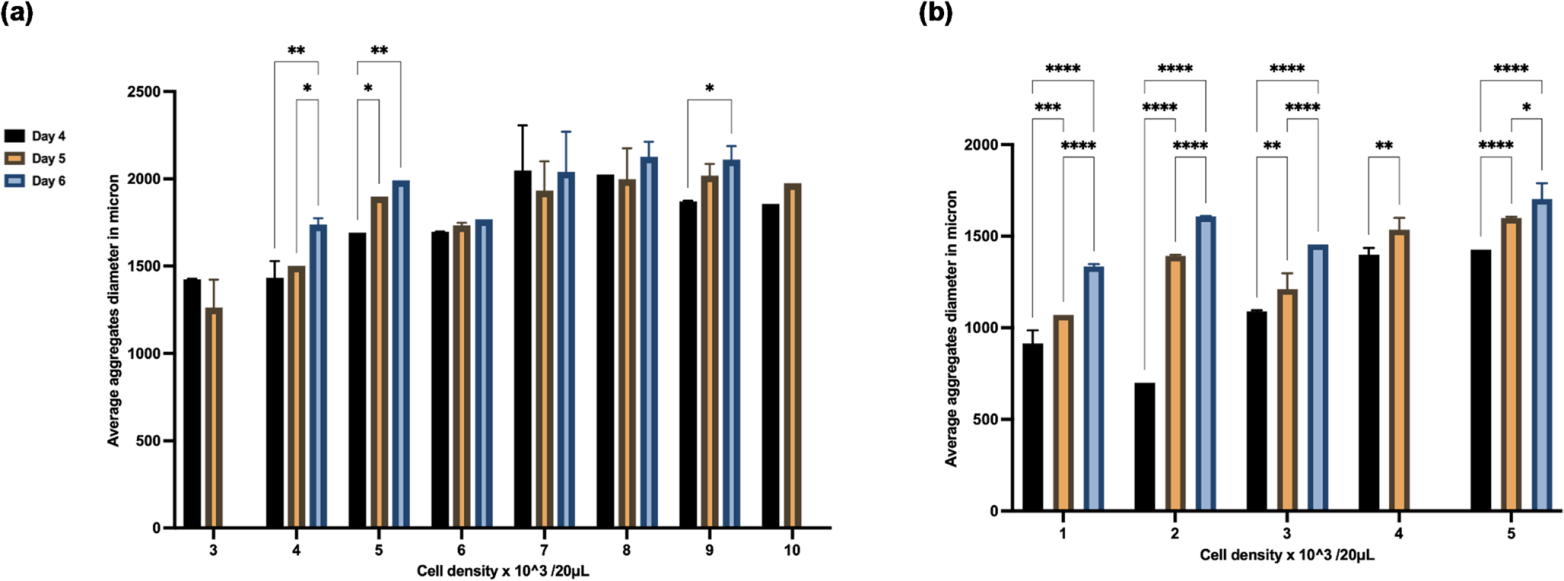
Three-dimensional (3D) culture developed from MDA-MB-468 TNBC cells [(1–10) × 10^³ cells/20 µL] cultured in complete medium on days 4, 5, and 6. **[a]** Normal oxygen condition (NOC) or **[b]** Low oxygen condition (LOC). The average change in diameters of 3D-culture was recorded on these days **[a]** NOC and **[b]** LOC. No spheroid aggregates formed at 1–2 × 10^³ cells/20 µL under NOC, while under LOC, at cell density > 5 × 10^³ cells/20 µL were unstable and 4 × 10³ cells/20 µL lost compaction by day 6. The best spheroid aggregates were observed when 4–5 × 10^³/20 µL under both conditions and 6 × 10^³/20 µL under NOC. Data are presented as the average percentage diameter ± standard deviation (SD) of from three independent biological replicates, with > 3 spheroids cell density. Statistical significance (two-way ANOVA), *p* < 0.05 (*) and *p* < 0.01 (**), *p* < 0.001(***), *p* < 0.0001(****). Images were captured using an inverted light microscope (Nikon Eclipse TE2000-U inverted light microscope)

**Fig. 3:**
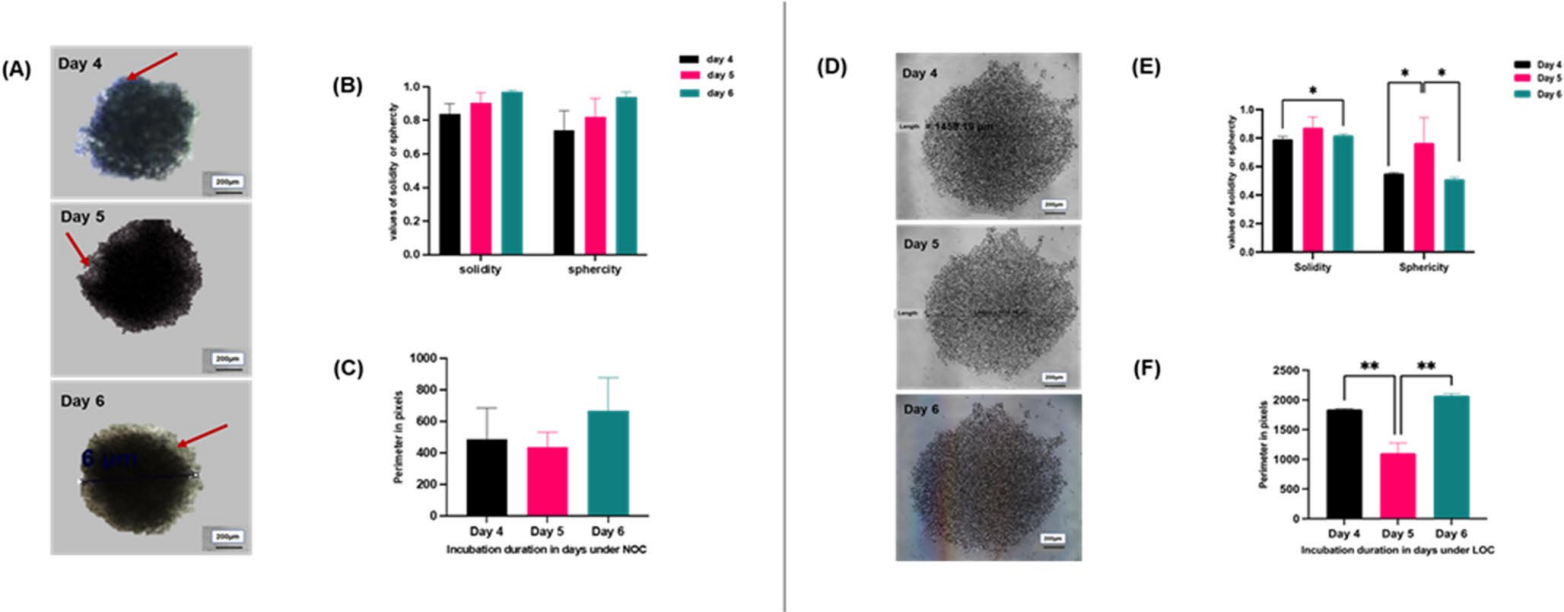
3D culture formation was assessed using agarose-coated 96-well plates under two oxygen conditions: normoxic (NOC) [A] and low oxygen conditions (LOC) [D]. Morphometric parameters, including solidity, sphericity, and perimeter, were quantified using AnaSP image analysis software. Results for spheroids formed under NOC are shown in panels [B] and [C], and under LOC in panels [E] and [F]. Images are representative of a single well that was followed longitudinally across the captured time period; Red arrows indicate cellular debris. Data are mean ± SEM from three independent biological replicates, with > 3 spheroids per condition. Statistical significance: *p* < 0.05 (*) and *p* < 0.01 (**) using one -way ANOVA. Images were captured using an inverted light microscope (Nikon Eclipse TE2000-U) at 4× magnification. **3D: three dimensional**; **NOC: normal oxygen condition; LOC: Low oxygen condition; scale bar = 200 micron**

**Fig. 4:**
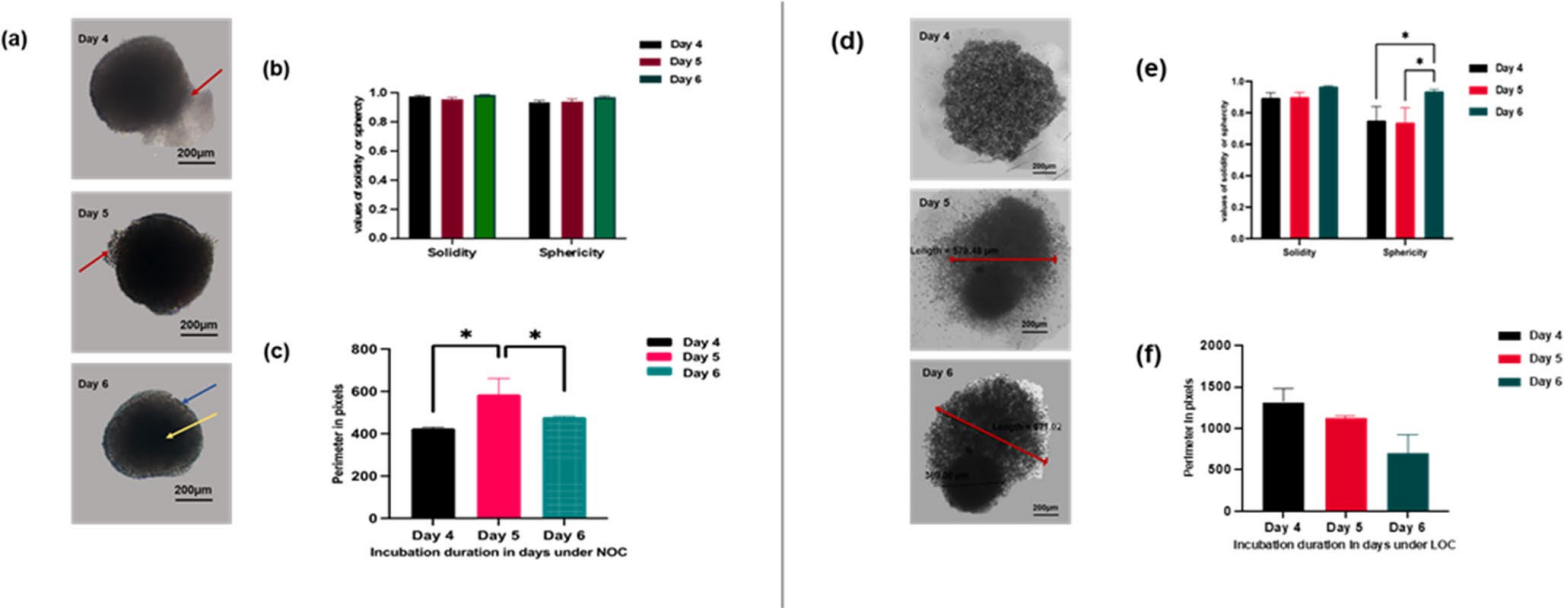
3D culture formation was assessed using ultra-low attachment (ULA) plates under normal (NOC) **[a]** and low oxygen conditions (LOC) **[d]**. Morphometric parameters, including solidity, sphericity, and perimeter, were quantified using AnaSP image analysis software. Results for spheroids formed under NOC **[b**,** c]** and LOC **[e**,** f]**. Images are representative of a single well that was followed longitudinally across the captured time period. Red arrows indicate cellular debris; the yellow arrow shows the core, while the blue arrow points to the spheroid’s outer layer. Data are expressed as the mean ± standard error of the mean (SEM) from three independent biological replicates. Statistical significance was determined as *p* < 0.05 (*) using one-way ANOVA. Spheroid images were captured using an inverted light microscope (Nikon Eclipse TE2000-U) at 4× magnification. **3D: three dimensional**; **NOC: normal oxygen condition; LOC: Low oxygen condition**,** scale bar = 200 micron**

Cells were harvested via trypsinisation, centrifuged, and resuspended in complete medium. Drops (20 µL) of various cell densities (1–10 × 10^3 cells) were pipetted onto the inner surface of the Petri dish lids (JET BIOFIL, Cat. No.754001, China). Dishes were inverted over PBS-filled bases to prevent evaporation and incubated at 37 °C under normal oxygen conditions (NOC; 19% O₂) or low oxygen conditions (LOC; 4% O₂) for 24–36 h (Figs. 1, 2, 3 & 4**)**. After 36 h, aggregates were transferred to 96-well plates (JetBiofil, Cat. No.CAP011096, China) pre-coated with 50 µL of 1% agarose per well [32], and overlaid with complete medium. Transfer at this time was carried out because a continued inverted culture becomes suboptimal as spheroids enlarge, risking droplet instability and diffusion limitations. This transfer also allowed for standardised medium renewal and downstream analyses within a non-adherent, well-based format. The medium was changed every two days. To minimise disturbance, plates were handled carefully; medium was slowly aspirated and dispensed along the well. “An ‘aggregate’ at a given time point was defined a priori as a cohesive, circular/elliptical body with a diameter ≥ 50 µm and sustained compactness for ≥ 36 h. Wells that formed transient clusters at 24–36 h but lost cohesion by day 6 were scored as ‘no aggregate’ at day 6.”

##### Liquid overlay

**Fig. 5:**
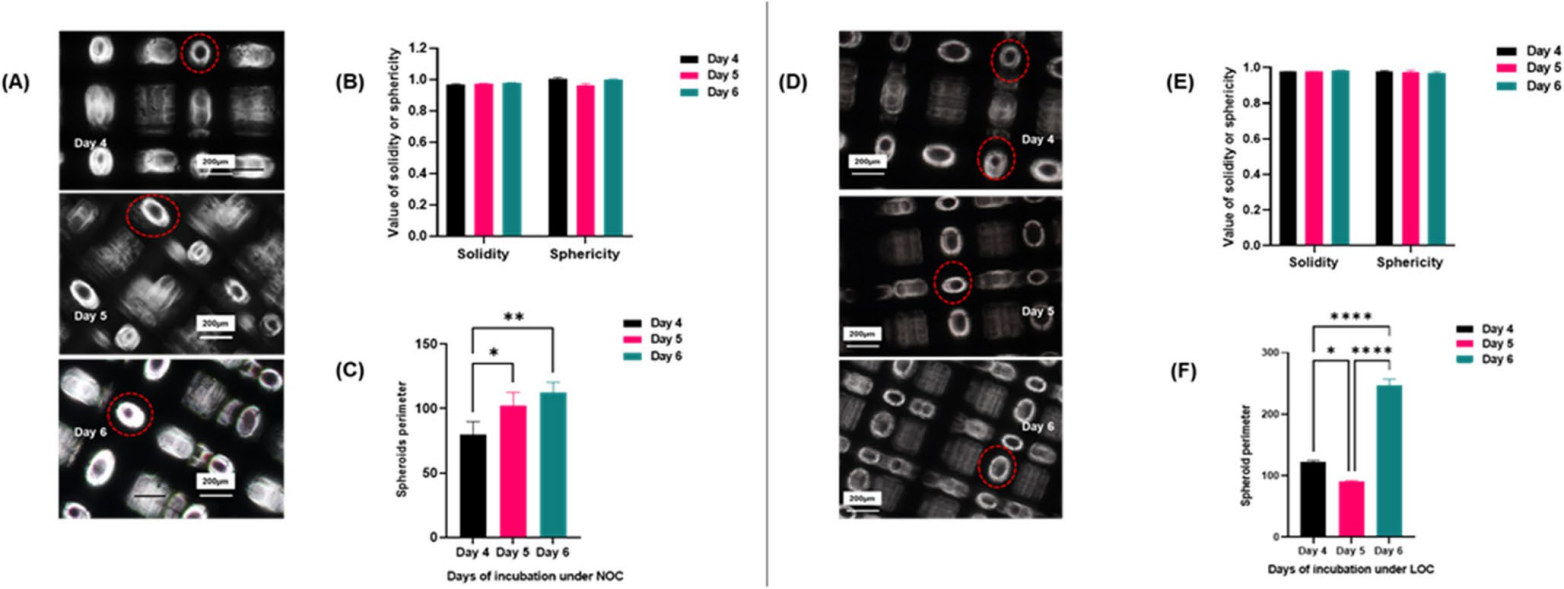
Series of spheroid images formed using scaffold plate under NOC **[A]** or low LOC **[D]**. AnaSP software solidity, sphericity and perimeter as detected by the software under NOC **[B and C]** and LOC **[E and F]**. Red circles indicate 3D cultures that met the inclusion criteria for analysis, as outlined in **Sect. 2.2.1.** Data is represented as the mean of three biological replicates ± standard error of the mean (SEM). Using one-way ANOVA, *p* < 0.05 (*) and *p* < 0.01 (**), *p* < 0.001(***), *p* < 0.0001(****). Images were captured using an inverted light microscope (Nikon Eclipse TE2000-U inverted light microscope). 4× magnification.D: three dimensional; NOC: normal oxygen condition; LOC: Low oxygen condition bar =200micron

**Fig. 6:**
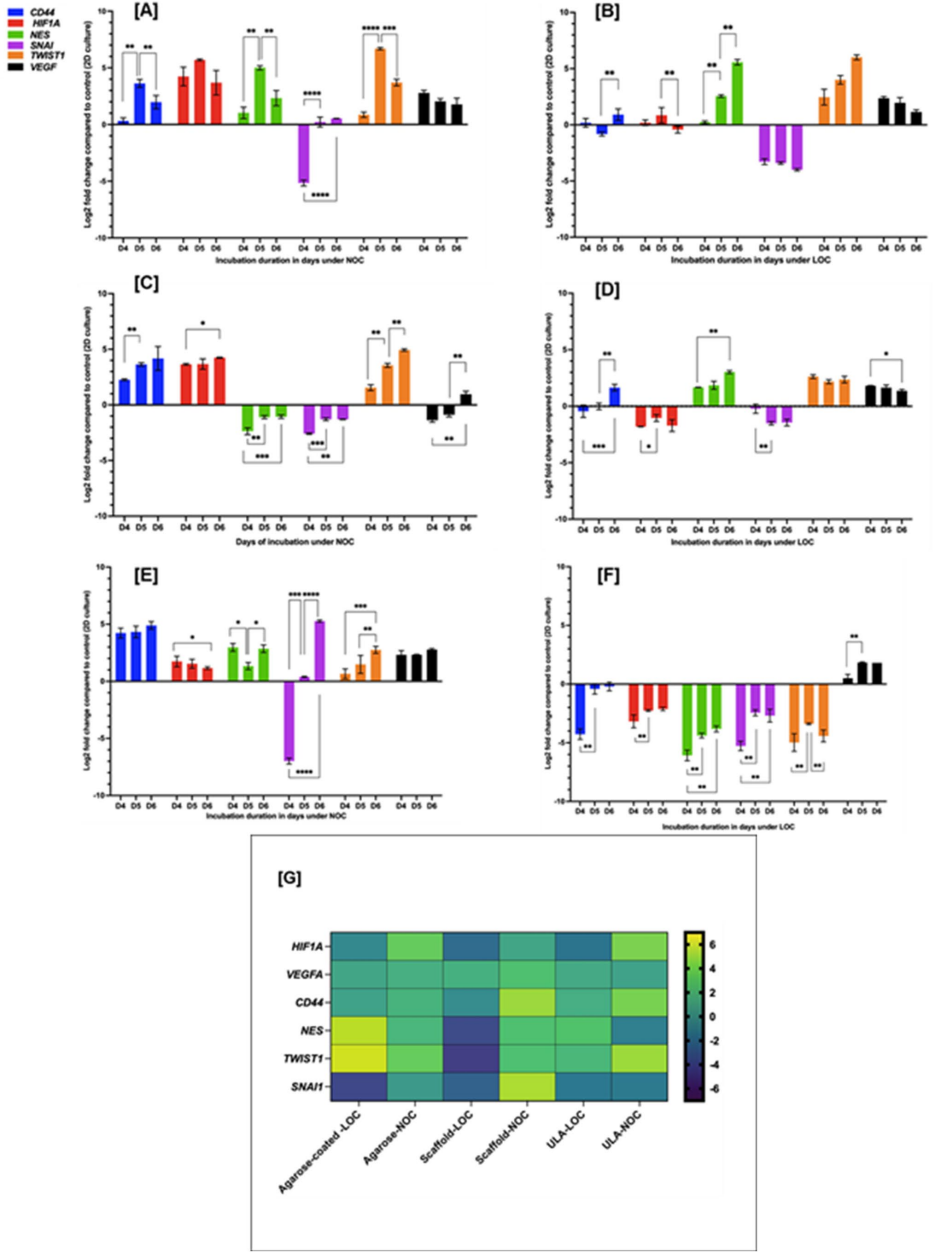
qPCR analysis of gene expression in 3D spheroids generated under normoxic and low-oxygen conditions. Relative mRNA levels of *CD44*, *HIF1A*, *NES*, *SNAl1*, *TWIST1* and *VEGFA* were quantified by qPCR in MDA-MB-468 spheroids formed using **[A - B]** agarose-coated 96-well culture plate, **[C- D]** ULA-plate and **[E**,** F]** scaffold plate under NOC [**A**, **C**, **E]** and LOC **[B**, **D**, **F].** Expression was assessed on days 4, 5, and 6 post-seeding and compared to 2D (control), **[G]**Day-6 heat map comparing log₂ expression of *CD44*, *HIF1A*, *NES*, *SNAI1*, *TWIST1*, and *VEGFA* across all conditions (agarose, ULA, scaffold; NOC and LOC). Colour scale: yellow = higher expression, purple = lower expression. Values are means of *n* = 3 biological replicates. Two-way or one-way ANOVA was applied, as appropriate. Statistical significance was set at *p* < 0.05 (*) and *p* < 0.01 (**), *p*< 0.001(***), *p*< 0.0001(****)**D=Days; ULA-plate: ultra-low attachment 96well plate; NOC: normal oxygen condition; LOC: Low oxygen condition**

Aggregates (cell density 5 × 10^3 cells/20 µL) were generated using the hanging-drop (HD) method and pre-cultured for 24–36 h to initiate spheroidity before being transferred into the non-adherent plates. Uniform aggregates were then placed into at the with same aggregates formed at the same time points with around 10 minuts difference for each replica (i) agarose-coated 96-well plates (JetBiofil, Cat. No.CAP011096, China) coated with 50 µL 1% agarose/well [32], or (ii) Ultra–low-attachment (ULA) 96-well plates (Corning^®^, Cat. No. 4515, Corning, USA). Cultures were maintained for six days under NOC or LOC (Figs. 1–5 & 6) in complete medium supplemented with 0.2% rat-tail collagen I (Cat. No. A10483-01, Gibco, USA; stock concentration 3–4 mg/mL; stored at 4 °C.) for six days under NOC or LOC. The medium changed every two days. To minimise mechanical disturbance, the medium was aspirated/dispensed slowly along the well wall. Collagen supplementation was not applied to the comparator methods.

#### Scaffold culture

The MDA-MB-468 cells were grown in T75 culture flasks containing the complete medium under NOC conditions until confluency. Single cell suspensions (1–10 × 10^3 cells/20 µL) were prepared. Then, Scaffold 6-well plates (CellSCAFLD^®^ 3D Cell Culture Scaffolds, Cat.No. TDP032006, JetBIOFIL, China) with 260–500 μm wire spacing were seeded with 1.5 mL of the cell suspensions and incubated under NOC or LOC conditions for six days.

#### Statistical analysis

Statistical analyses were performed using GraphPad Prism v9.5.1 for Windows (GraphPad Software, San Diego, CA, USA, www.graphpad.com). Spheroid morphology data were analysed using two-way ANOVA when comparing multiple factors (e.g., method × time/oxygen) and one-way ANOVA for single-factor comparisons. For repeated-measures designs, sphericity was not assumed; Greenhouse–Geisser (GG) corrections were applied to the degrees of freedom, and GG-corrected p-values were reported. Unless specified otherwise, all tests were two-sided with α = 0.05. Data are presented as mean ± standard error of the mean SEM (or mean ± standard deviation SD, where indicated). Each experiment was performed at least three times, with a minimum of three samples per experiment.

### Characterisation of MDA-MB-468 spheroid model

#### Bright-field microscopy and AnaSP software analysis

Spheroids were visualised using a Nikon Eclipse TE2000-U inverted microscope. Image analysis was conducted with AnaSP software (v2.0), integrated with MATLAB R2022a [33]. The standard parameters extracted using the software included Area, Convexity, Perimeter, Solidity, Sphericity, and Volume [33, 34]. At each time point and for every cell density/condition, 5–6 spheroids per biological replicate were imaged (*N* = 3), yielding *n* = 15–18 spheroids per density per time point. Image-derived metrics were quantified using AnaSP. Unless otherwise specified, all images satisfying a priori quality criteria, adequate focus and reliable boundary segmentation were included in the analysis. For rigid scaffold culture images were captured at 4× magnification at 24-hour intervals, and data from days 4 to 6 were used for analysis. Spatial calibration was derived from the microscope scale bar and was consistently applied to the images. AnaSP was employed to calculate the equivalent diameter, perimeter, circularity, and solidity from binarised masks. The inclusion criteria were as follows: (i) a complete, closed contour fully contained within the frame with no edge contact; (ii) no overlap or merger with neighbouring objects or scaffold struts; and (iii) sufficient focus for boundary detection across three or more adjacent z-planes. The exclusion criteria included edge-touching or truncated objects, overlapping or tethered aggregates, and images that were out of focus. Consequently, quantitative summaries reflect only fully imaged spheroids or compact aggregates, and representative figure panels are cropped from full-field acquisitions.

#### Gene expression analysis

##### Cell seeding and normalisation for gene expression:

Monolayers/2D MDA-MB-468 TNBC were seeded for gene expression experiments to match the 3D cell-per-surface-area burden. Using 5 × 10^³ cells per 20 µL hanging drop with a ≈ 5 mm footprint (area ≈ 0.196 cm²), the matched density was ≈ 2.53 × 10^⁴ cells/cm². Accordingly, T75 flasks (75 cm²) were seeded with ≈ 1.9 × 10^⁶ cells (equivalent to 1.9 × 10^⁵ cells/mL in 10 mL of complete media). 2D cells (control) were harvested or sub-cultured on day 3 following ATCC protocol and served as a control.

##### RNA isolation and qPCR:

Total RNA was isolated using the RNeasy Lipid Tissue Kit (QIAGEN, Hilden, Germany). RNA quality was assessed using NanoDrop (Implen, USA). Reverse transcription and quantitative polymerase chain reaction (qPCR) were performed using gene-specific primers *CD44*, *HIF1A*, *NES*, *SNAI1*, *TWIST1*, and *VEGFA* (Supplementary Information (SI) Table 1) purchased from NextGene Scientific (Petaling Jaya, Malaysia). GAPDH was used as a housekeeping gene. Relative expression was calculated using the 2^-ΔΔCT method [35]. Triplicate samples were analysed using GraphPad Prism. Student’s t-test and ANOVA with Greenhouse–Geisser correction were used for the statistical analysis.

#### Field emission scanning electron microscopy

Spheroids (MDA-MB-468) from ULA-NOC culture (day 6) were fixed in 4% formalin and incubated at 4 °C for one h. Subsequently, the samples were washed with PBS, dehydrated through graded ethanol, and visualised using a Hitachi SU8010 FE-SEM to investigate the ultrastructural surface changes.

#### Fluorescence microscopy

On the sixth day of culture, live spheroids (MDA-MB-468) from ULA-NOC cultures were subjected to staining with Hoechst 33,342 at a concentration of 1–2 µg/mL in a complete medium. The spheroids underwent a one-hour incubation, followed by two washes with PBS. In parallel, spheroids from the same cultures were fixed using 4% formalin and stained with the same concentration of Hoechst 33,342. Imaging was performed using a widefield fluorescence microscope (Olympus, Japan), with excitation at 386 nm and magnifications of 4× and 10×. Exposure times were optimised per sample to avoid signal saturation. The fluorescence intensity and dye penetration analysis were performed using ImageJ [36] to assess the viability and morphological characteristics of the spheroids.

## Results

The inclusion and exclusion criteria were used across all 3D spheroid culture methods to ensure reliable morphometric analysis. In the HD model, only aggregates ≤ 5 μm in diameter, entirely contained within the droplet, were transferred; aggregates were observed for 6 days; aggregates that maintained cohesive cellular organisation for at least 48 h were included, while edge-touching or fragmented aggregates were excluded. Only centrally located, intact spheroids with visible contours were included in the liquid-overlay method, while merged structures were omitted. Imaging was performed in situ for scaffold cultures to prevent disturbance, and only spheroids with continuous cellular boundaries traceable across ≥ 3 z-planes were accepted. Across all methods, a minimum equivalent diameter of 50 μm and clear boundary definition were required to define spheroid formation. During longitudinal tracking (days 4,5, and 6), only spheroids remaining entirely within the imaging frame were analysed, ensuring that quantitative measurements represented morphologically stable 3D structures.

### Hanging drop

#### HD spheroids characterisation using bright field microscopy

The HD method is based on the concept of forming homogeneous multicellular tumour spheroids by inducing cell aggregation via gravity to facilitate cell settling, adhesion, and subsequent aggregate formation. A series of images of MDA-MB-468 3D cultured aggregates under NOC or LOC was captured on days four, five, and six post-seeding **(Supplementary information (SI) 1)**. Diameter measurements using ImageJ were analysed to determine the optimal cell density for reproducible spheroid formation. The measurement results indicated a distinct variation in spheroid aggregate diameter across different days and cell densities under the NOC and LOC (Fig. 2). When comparing various seeding densities, the images revealed that both the aggregate density and diameter increased generally in tandem with the cell density. However, this increase did not correspond to an increase in core density or maturity of the layers within the respective spheroid aggregates.

The average diameter of spheroid aggregates was analysed at various time points, showing that certain cell densities did not result in the formation of spheroid aggregates. For instance, no maintainable aggregates were formed at 1–2 × 10^³ cells/20 µL under NOC, and under LOC spheroids > 5 × 10^³ cells/20 µL (Fig. 2b). Also, the cell densities under 4 and 10 × 10^³ cells/20 µL (NOC) lost compaction by day 6, suggesting that early aggregates were detectable at 24–36 h but failed to maintain compactness by day 6 in the absence of ECM (Fig. 2a). This observation may be attributed to the manual handling of the plates, which could have disrupted the organised circular arrangement of cellular aggregates. It may also be specific to the MDA-MB-468 cell line, as reported by Gencoglu et al. [37]. Overall, seeding densities of 5 × 10^3 cells/20µL and 6 × 10^3 cells/20 µL demonstrated optimal results under NOC with minimal standard deviation. Also, the 5 × 10^3 cells/20µL density exhibited statistically significant variations in aggregate size across different days, with a p-value < 0.05. Comparable patterns were observed under LOC. Cellular aggregates formed using the HD method generally exhibited variable diameters depending on the cell density and oxygenation. An additional HD experiment was conducted using MDA-MB231 TNBC cells (**SI5-6**).

### Liquid overlay

Direct seeding of 5 × 10^3/20 µL into agarose-coated or ULA-plates did not produce spheroids. More compact spheroid formation was observed only when 0.2% rat-tail collagen I was incorporated into the medium. In its absence, MDA-MB-468 aggregates remained loose and failed to maintain structural integrity (loose aggregates), confirming that ECM support is required for stable spheroid maturation in liquid overlay. A standard seeding density of 5 × 10^3 cells per 20 µL, grown in complete medium supplemented with 0.2% rat-tail collagen.

#### Characterisation using bright field microscopy and AnaSP software analysis

##### Agarose-coated 96-well culture plate under normal or low oxygen conditions

The images of MDA-MB-46 3D spheroids was obtained during this timeframe (days 4, 5, and 6) revealed that the aggregates formed more compact, circular structures with a dark core, of variable sizes and shapes under NOC (Fig. 3A). In contrast, the cells that developed in the LOCs appeared less compact and larger in size(Fig. 3D). Quantitative analysis using AnaSP, sphericity and solidity values of spheroids, derived from agarose-coated 96-well culture plates, increased progressively over the incubation period under NOC (Fig. 3B). Notably, this increase did not reach statistical significance across the evaluated time points, contrasting with the significant variations observed under LOC (Fig. 3E). At the same time, the perimeter of the 3D culture displayed a variable pattern each day, with the most substantial increase observed on day 6 under both oxygenation conditions (Fig. 3C **and F**).

##### Ultra-low attachment 96-well plate normal or low oxygen conditions

MDA-MB-468 3D spheroids formation became apparent by the fourth day of culture, with the most well-developed structures observed on day six (Fig. 4a). The spheroids displayed a compact morphology, characterised by two distinct zones: a lighter outer zone and a denser inner zone, consistent with the appearance of compact spheroids. This zonal structure was particularly evident in the NOC condition on day six, where the outer region appeared darker, and the centre was noticeably denser (Fig. 4d). In contrast, spheroids formed under LOC conditions were smaller and surrounded by substantial cellular debris. AnaSP analysis revealed that spheroids formed under NOC conditions maintained high sphericity and solidity values, both approaching 1 across all assessed time points, with no significant variations (Fig. 4b). Notably, these values were not observed under LOC conditions until day six (Fig. 4e). In addition to that, parameters such as sphericity and diameter of spheroids generated using ULA-plates under NOC conditions exhibited minimal standard deviation across replicates, with a similar trend observed for perimeter measurements, except on day five (Fig. 4).

### Scaffold culture

Rigid scaffold cultures were imaged in situ using bright-field microscopy to prevent mechanical disruption associated with scaffold washout. Consequently, quantitative analyses were conducted using non-destructive morphometrics (area, diameter, and circularity) calculated using AnaSP. Representative panels include day 0 of single-cell seeding, and 10× magnified insets with boundary overlays and scale bars. The scaffold materials exhibited intrinsic fluorescence, which restricted the use of conventional green/red channel imaging. Irrespective of the seeding density employed (1–10 × 10^3 cells/20 µL), spheroids formed within 24–48 h, demonstrating consistent spheroid formation across several densities (**SI 2–4**). Varying sizes of spheroids were observed under both oxygenation conditions (**SI2**). The number and size of spheroids per microscopic field remained consistent across different incubation durations and morphologically were related to the cellular density per mL (**SI 2**).

A cell density of 5 × 10^3 cells/20 µL was standardised to ensure consistency in comparing culture methods and facilitate a more precise evaluation of the effectiveness of different culture techniques. The scaffold spheroids were subsequently examined using a bright-field microscope, with images captured every 24 h over the fourth, fifth, and sixth post-seeding. Sizes ranged from 150 to 50 μm. AnaSP analysis showed constant sphericity and solidity regardless of oxygen level or time point (Fig. 5B and E). Under NOC, the perimeter steadily increased; under LOC, the perimeter increased on day 6 (*p* < 0.0001) but was notably reduced on day 5 (*p* < 0.05) compared to day 4, suggesting instability or possible disaggregation(Fig. 5C and F). Generally, spheroid formation using scaffold-based 3D culture was uncomplicated and yielded spheroids at various time points. Certain challenges were encountered during the process, including (i) precise targeting and isolation of single spheroids within the porous architecture proved difficult, and (ii) Accurate enumeration was hindered by optical artefacts generated by the synthetic scaffold. Additionally, these challenges are made more difficult by the limited resolution of traditional microscopes.

### Gene expression analysis

The shift from two-dimensional (2D) cell culture to spheroid formation was further explored by evaluating the expression levels of six genes associated with stemness, EMT, and hypoxia: *CD44*, *HIF1A*, *NES*, *SNAI1*, *TWIST1*, and *VEGFA*. The expression of these genes was quantified in 3D cultures that were cultivated on days 4, 5, and 6 under NOC and LOC using three methodologies: agarose-coated 96-well plates (Fig. 6A **and B**) (ULA) plates (Fig. 6C **and D**), and scaffold systems (Fig. 6E **and F**). The expression of CD44 was significantly upregulated in spheroids grown under NOC with *p* ≤ 0.05, in liquid overlay methods, as shown in **(**Fig. 6A, C, **and E)**. The upregulation of *CD44* suggests the induction of stemness in 3D culture. The same pattern was observed in spheroids grown under LOC conditions on day 6 (Fig. 6B, D, **and G**) except in the scaffold culture (Fig. 6F**)**. The expression of the *HIF1A* gene was elevated in all spheroids cultivated under NOC, and with significant change observed in ULA-plate culture between day 4 and 6 (its expression through the examined days, however in the scaffold culture there was apparent significate change of the *HIF1A* expression between day 4 and day 6, which showed a significant decrease (*p* ≤ 0.05) (Fig. 6E). While it was under-expressed in spheroids cultured under LOC (Fig. 6B, D **and F**), except for spheroids developed on agarose-coated 96-well plates on days 4 and 5 (*p* ≤ 0.01) (Fig. 6B).

The *VEGFA* gene was upregulated in all spheroid cultures on days 4, 5, and 6 (Fig. 6), except for the spheroids cultured using the ULA-plate under NOC; its expression was downregulated on days 4 and 5, contrary to day 6, where it was significantly upregulated (*p* ≤ 0.01) (Fig. 6C). However, there was no overall reduction in *VEGFA* expression across the sixth day expressions (Fig. 6G). Interestingly, *NES* expression increased in spheroids developed using an agarose-coated 96-well plate under both oxygenation conditions (Fig. 6A **and B**). It was upregulated in spheroids formed with rigid scaffold plates under NOC, downregulated under LOC (Fig. 6E **and F**); the opposite was detected in ULA-plate under LOC (Fig. 6C **and D**).

In most of the examined culture development methods, *SNAl1* was under-expressed compared to 2D culture, except for those generated in agarose-coated 96-well plates on days 5 and 6 under NOC (*p* ≤ 0.0001) **(**Fig. 6A**)**, as well as in scaffold culture on days 5 and 6 (*p* ≤ 0.0001) under NOC **(**Fig. 6E**)**. While the expression of *SNAl1* was reduced in the spheroids generated from the ULA-plate under NOC, there was a significant downregulation between days 4 and day 6 (*p* ≤ 0.01), with day 5 having a lower expression (*p* ≤ 0.0001) **(**Fig. 6C**)**. Another EMT regulator *TWIST1* was substantially increased in expression across all spheroid cultures (Fig. 6), except for those generated using the scaffold method under LOC **(**Fig. 6F**)**.

In examining various culture methods and conditions, notable differences in gene expression levels were identified, signifying a shift towards spheroid culture. Collectively, these transcriptional changes corroborate the morphological progression seen in earlier figures (Figs. 2, 3, 4 and 5), where compact spheroids formed predominantly under NOC.

The data advocate that normoxic conditions promote spheroid maturation and the activation of stress-adapted EMT-related gene signatures. In contrast, hypoxic conditions disrupt cellular compaction (compact aggregates) and produce heterogeneous expression profiles. **(**SI Table 2**)** summarises and compares the morphological and molecular outcomes of different 3D culture platforms. Among these, the ULA-plate under NOC produced the most uniform and compact spheroids (precisely, day 6), making it the optimal model for downstream analyses. To further validate this approach, the same culture method was applied to another TNBC cell line, MDA-MB-231, and corresponding morphological changes were quantified using AnaSP image analysis **(**SI. 7**)**.

### Field emission scanning electron microscopy (FE-SEM)

**Fig. 7:**
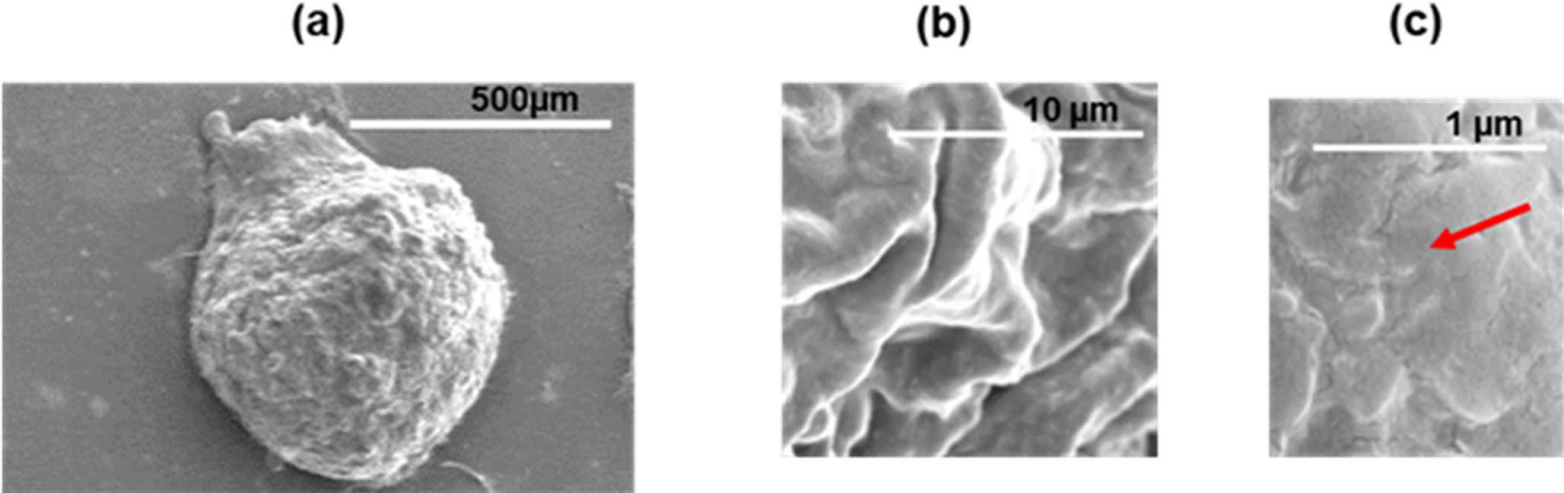
Morphological and ultrastructural characterization of MDA-MB-468 spheroids cultured in ultra-low attachment (ULA) plates under normal oxygen conditions (NOC) on day 6, **[a]** well-defined spherical morphology at (45×) magnification **[b]** visible surface invaginations and protrusions at (5000×) magnification **[c]** at ultra-high magnification (30,000×), detailed structural resolution reveals tight intercellular junctions and uniform surface texture, consistent with mature spheroid formation marked (red arrow).**ULA-plate: ultra-low attachment 96well plate; NOC: normal oxygen condition; Scale bar: (a): 500 μm; (b); 10 μm (c)1 μm**

To assess the microarchitecture of day 6 - ULA-Plate spheroids formed under NOC, FE-SEM; model SU8000 was employed at an accelerating voltage of 5.0 kV. At low magnification (45×), the spheroids appeared nearly spherical with smooth, well-defined boundaries, suggesting organised cell-cell interactions (Fig. 7a). At a higher magnification (5000×), the spheroid surface displayed fine structural features, including localised invaginations and cellular protrusions (Fig. 7b). At ultra-high magnification (30,000×), a detailed examination revealed uniform, tightly packed cellular junctions signifying strong intercellular adhesion (Fig. 7c). These features are critical for maintaining the cohesion and structural integrity of the spheroids indicating strong adhesion and structural maturity.

### Fluorescence microscopy

**Fig. 8:**
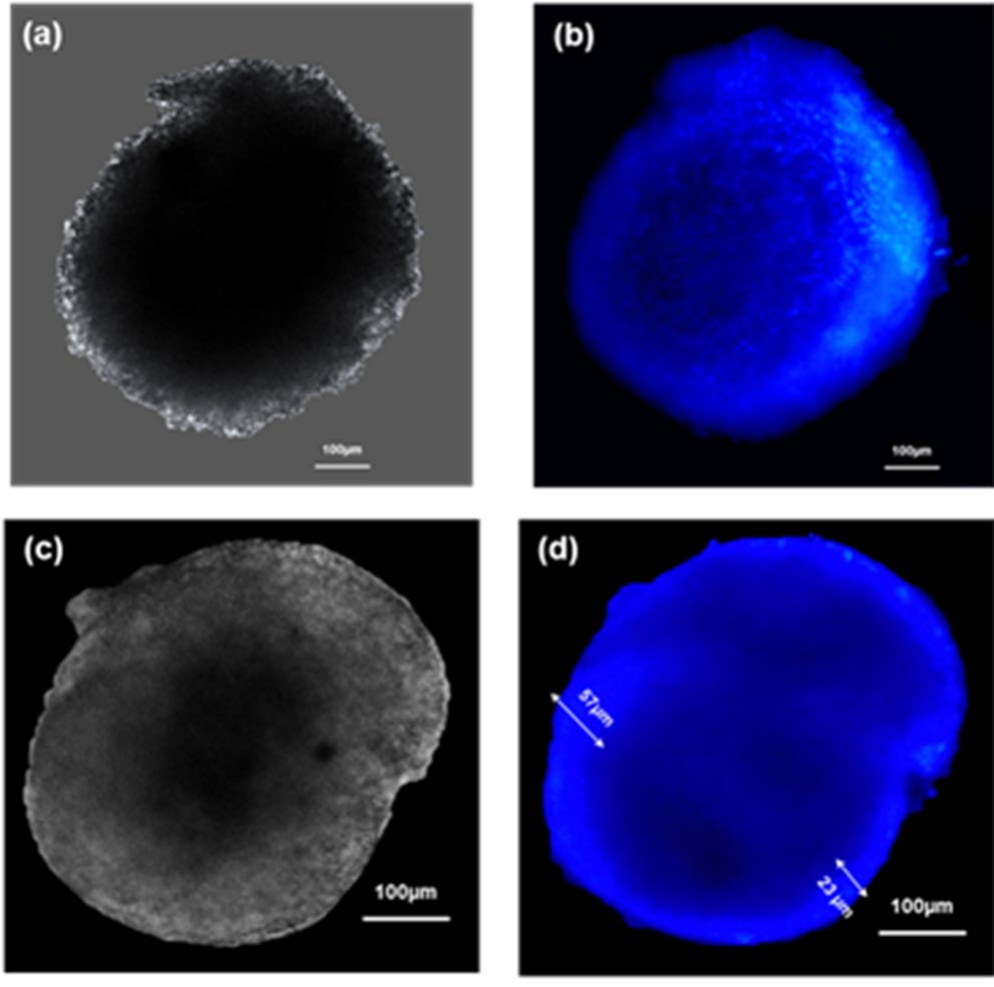
day 6 NOC-ULA-plate spheroid. **[a]** Bright-field image of a live spheroid showing intact, spheroidal morphology. **[b]** Fluorescence image of a live spheroid stained with Hoechst 33,342 (1–2 µg/mL, 1 h), showing uniform nuclear fluorescence throughout the spheroid, reflecting the dye permeability in viable cells. **[c]** Bright-field image of a fixed the fixed spheroids showing intact, spheroidal morphology. **[d]** Fluorescence image of a fixed spheroid stained with Hoechst 33,342 (1–2 µg/mL, 1 h) showing nuclear fluorescence confined to the peripheral cell layers (~ 23–57) µm, suggesting limited dye penetration. Images were acquired using a widefield fluorescence microscope (excitation: 386 nm) and analysed in ImageJ. **ULA-plate: ultra-low attachment 96well plate; NOC: normal oxygen condition; Magnification = 10x**; **Scale bar = 100 μm**

Day 6 - ULA-Plate spheroids developed under NOC were stained with Hoechst 33,342 (1–2 µg/mL) and imaged using fluorescence microscopy to evaluate nuclear dye penetration in both fixed and live 3D tumour models. Bright-field imaging confirmed the samples’ structural integrity and spheroidal morphology (Fig. 8a and c).

Fluorescence imaging of fixed spheroids (Fig. 10b) revealed a peripheral ring of Hoechst staining, with penetration depths ranging from approximately 23 μm to 57 μm from the spheroid edge. The fluorescence signal intensity diminished sharply toward the core, indicating limited diffusion of the dye in the fixed matrix, suggesting a barrier to molecular penetration likely due to fixation-induced crosslinking and dense cellular packing. The same was examined in live spheroids (Fig. 8a and b), which exhibited a significantly homogeneous fluorescence throughout the spheroid volume. The nuclear dye effectively penetrated the inner regions, indicating the permeability of the viable cells. The live day 6 ULA-plate spheroids staining pattern indicated that live-cell staining may facilitate deeper dye distribution, preserving some of the nuclear definition, while maintaining overall spheroid morphology.

## Discussion

In this study, direct seeding into liquid overlay platforms proved ineffective, consistent with the literature indicating that MDA-MB-468 cells exhibit weak intrinsic aggregation ability due to low E-cadherin expression and mesenchymal traits [38]. However, transferring HD-formed aggregates into ULA or agarose-coated plates supported maturation into spheroids with high solidity and sphericity values, particularly under NOC, likely due to stable oxygen availability and reduced environmental stress [39, 40]. In contrast, scaffold-based cultures facilitated rapid spheroid formation but exhibited increased heterogeneity and inconsistent gene expression patterns under hypoxic conditions. Physical interference from synthetic scaffold materials may disrupt cell-cell interactions [41], causing a reduction in sphericity and complicating spheroid isolation and imaging [42]. Despite their early and consistent formation, scaffold-grown spheroids experienced disaggregation and diminished structural integrity under LOC.

The study also demonstrated that the oxygen microenvironment and 3D architecture jointly regulate MDA-MB-468 TNBC spheroid phenotype and gene expression. The consistent upregulation of *CD44* and *TWIST1* across all NOC models underscores that spheroid formation promoted stemness and EMT expression characteristic of aggressive TNBC subtypes. These findings concord with established roles of CD44 metastatic potential [21] and TWIST1 in sustaining Mad-Mb-468 3D formation, growth, and chemoresistance [43].

The noticeable elevation of *HIF1A* under normoxia supports the concept of metabolic or pseudo-hypoxia, whereby intracellular stress stabilises *HIF1A* despite ambient oxygen. Similar effects have been reported in compact tumour spheroids [44]. That may arise from limited oxygen diffusion into spheroid cores and high cellular respiration, producing localised hypoxic zones and secondary *VEGFA* activation. The persistent upregulation of VEGFA across models (Fig. 6G) further substantiates the onset of an angiogenic angiogenesis, a hallmark of early tumour progression and a key feature of TNBC aggressiveness.

The expression of *SNAI1* and *NES* demonstrated variability that was specific to the method employed. While *NES*, a marker of neural stemness [45], was consistently elevated in agarose-plate cultures, its expression decreased under LOC in scaffolds, implying differential hypoxic stress responses and altered differentiation cues dependent on the matrix [46, 47]. The suppression of *SNAI1* in ultra-low attachment (ULA) cultures under NOC may reflect reduced EMT activation [48], possibly associated with the 3D spheroids’ compactness and phenotype.

These findings emphasise the significant impact of the culture platform and oxygenation on the development of MDA-MB-468 TNBC spheroids. Although scaffold-based systems enabled relatively rapid spheroid formation, their practical limitations, such as imaging challenges and variability in spheroid size, restricted their reliability for high-fidelity modelling (Fig. 5). In contrast, the combined hanging-drop (HD) to liquid-overlay approach, particularly using ULA-plates under NOC, produced spheroids that were both structurally consistent (Fig. 4a-c) and biologically relevant, exhibiting key features of stemness, EMT plasticity, and pseudo-hypoxia (Fig. 6c-d). Consequently, this model was considered the most suitable for preclinical drug-screening applications in this study setting. To validate this selection, only the ULA–NOC spheroids were subjected to detailed structural characterisation using FESEM and Hoechst nuclear staining to confirm spheroid integrity and compactness.

This study addressed some of the increasing demand for standardised TNBC cell lines, specifically MDA-MB-468 spheroid culture models, and suggests that adjusting oxygen levels and culture substrates is crucial for maintaining their physiological relevance.

## Conclusion

This study explored how different 3D culture methods and oxygen levels influence the formation and biological features of TNBC 3D spheroids derived from MDA-MB-468 cells. Among all methods, the hanging-drop followed by the liquid-overlay method, especially when using ULA-plates under normoxic conditions, produced structurally uniform and biologically relevant spheroids. These spheroids consistently showed expression of genes related to stemness, hypoxia adaptation, and EMT, making them suitable for modelling tumour behaviour and testing therapeutic responses. Importantly, preliminary drug-response studies performed during our doctoral research (unpublished data) have already shown that these spheroids produce reproducible therapeutic profiles, supporting their value as a translational platform for anticancer drug testing.

In the longer term, this model could provide a strong basis for developing more complex systems that incorporate co-culture conditions, tumour-on-chip devices, and dynamic flow environments to more closely replicate the tumour microenvironment. Furthermore, enhancing structural and imaging quality through far-red nuclear and cytoplasmic labelling for all investigated methods.

## Supplementary Information

Below is the link to the electronic supplementary material.

Supplementary Material 1

Supplementary Material 2

Supplementary Material 3

Supplementary Material 4

## Data Availability

The work presented in this manuscript forms a chapter in the PhD thesis of the first author. Data are provided in supplementary file.
