# Supplementary material for: Optimising the method to develop spheroids from MDA-MB-468 human triple negative breast cancer cells
Source: Mol Biol Rep. 2026 Jan 24;53(1):322. doi: 10.1007/s11033-026-11451-4 (PMC12831690; doi:10.1007/s11033-026-11451-4)
Supplement: Supplementary file 1 — Supplementary Material 1 [file 11033_2026_11451_MOESM1_ESM.pptx]

## Slide 1
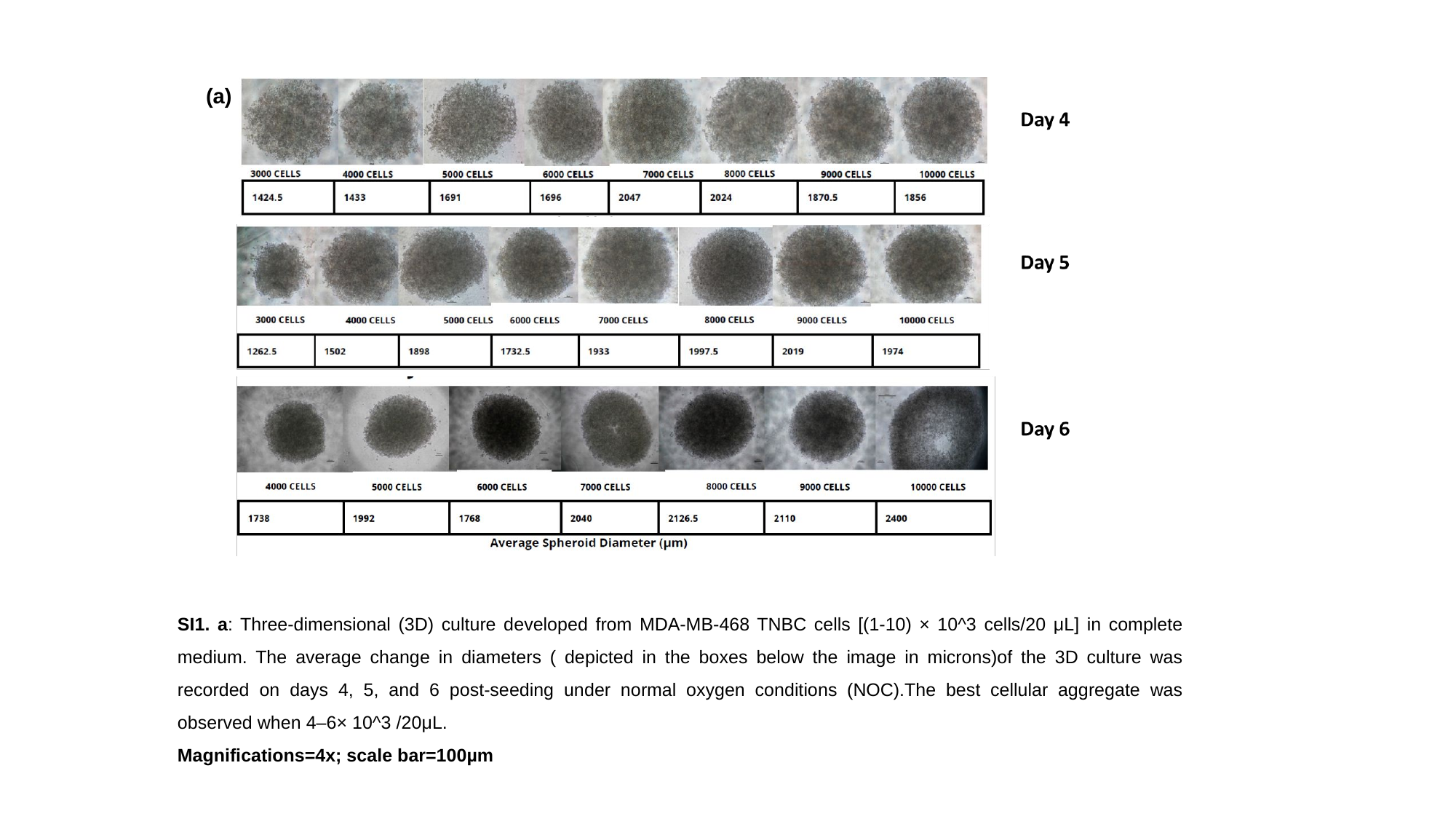

(a)
SI1. a: Three-dimensional (3D) culture developed from MDA-MB-468 TNBC cells [(1-10) × 10^3 cells/20 μL] in complete medium. The average change in diameters ( depicted in the boxes below the image in microns)of the 3D culture was recorded on days 4, 5, and 6 post-seeding under normal oxygen conditions (NOC).The best cellular aggregate was observed when 4–6× 10^3 /20μL.
Magnifications=4x; scale bar=100µm

## Slide 2
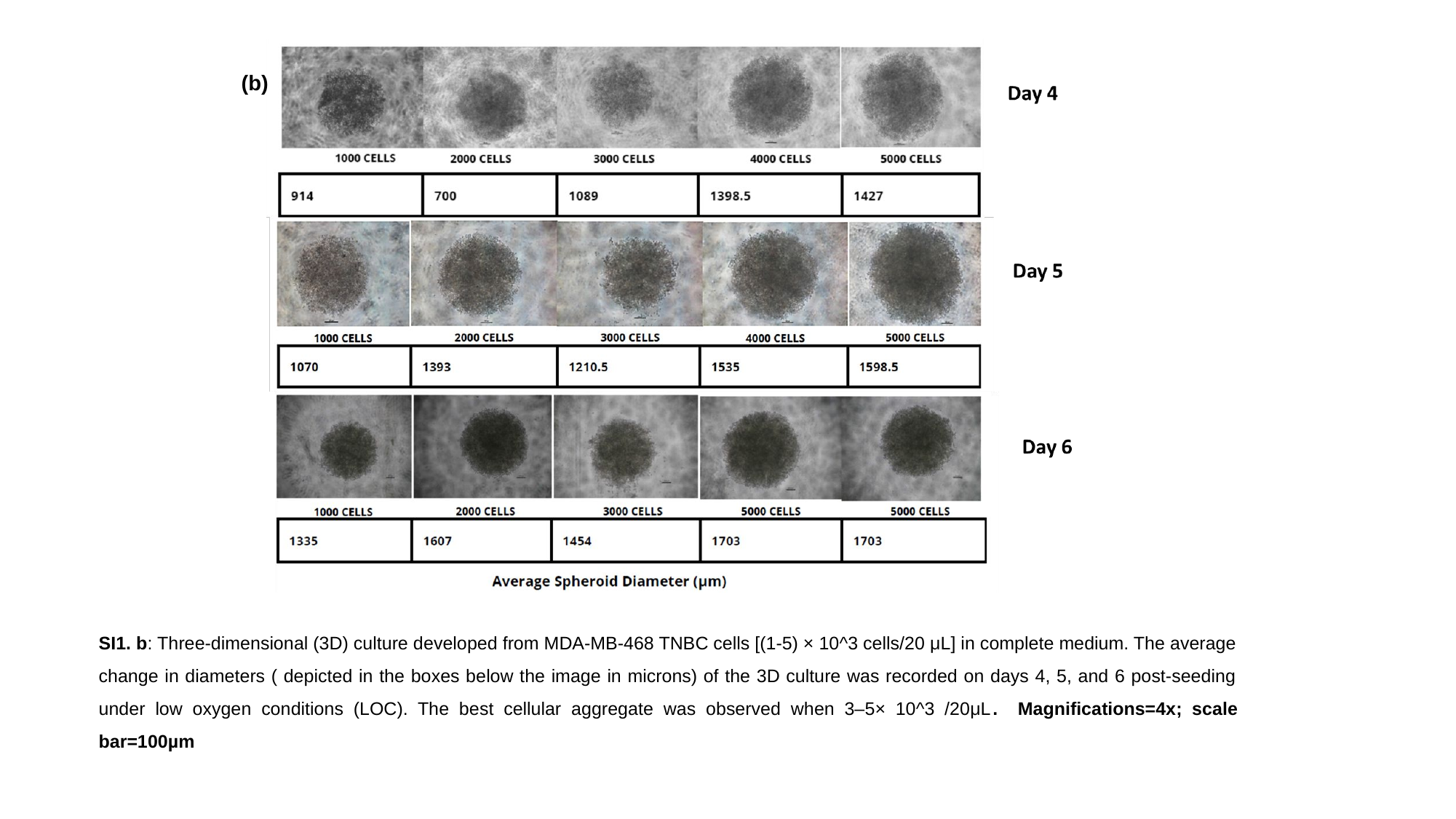

(b)
SI1. b: Three-dimensional (3D) culture developed from MDA-MB-468 TNBC cells [(1-5) × 10^3 cells/20 μL] in complete medium. The average change in diameters ( depicted in the boxes below the image in microns) of the 3D culture was recorded on days 4, 5, and 6 post-seeding under low oxygen conditions (LOC). The best cellular aggregate was observed when 3–5× 10^3 /20μL. Magnifications=4x; scale bar=100µm

## Slide 3
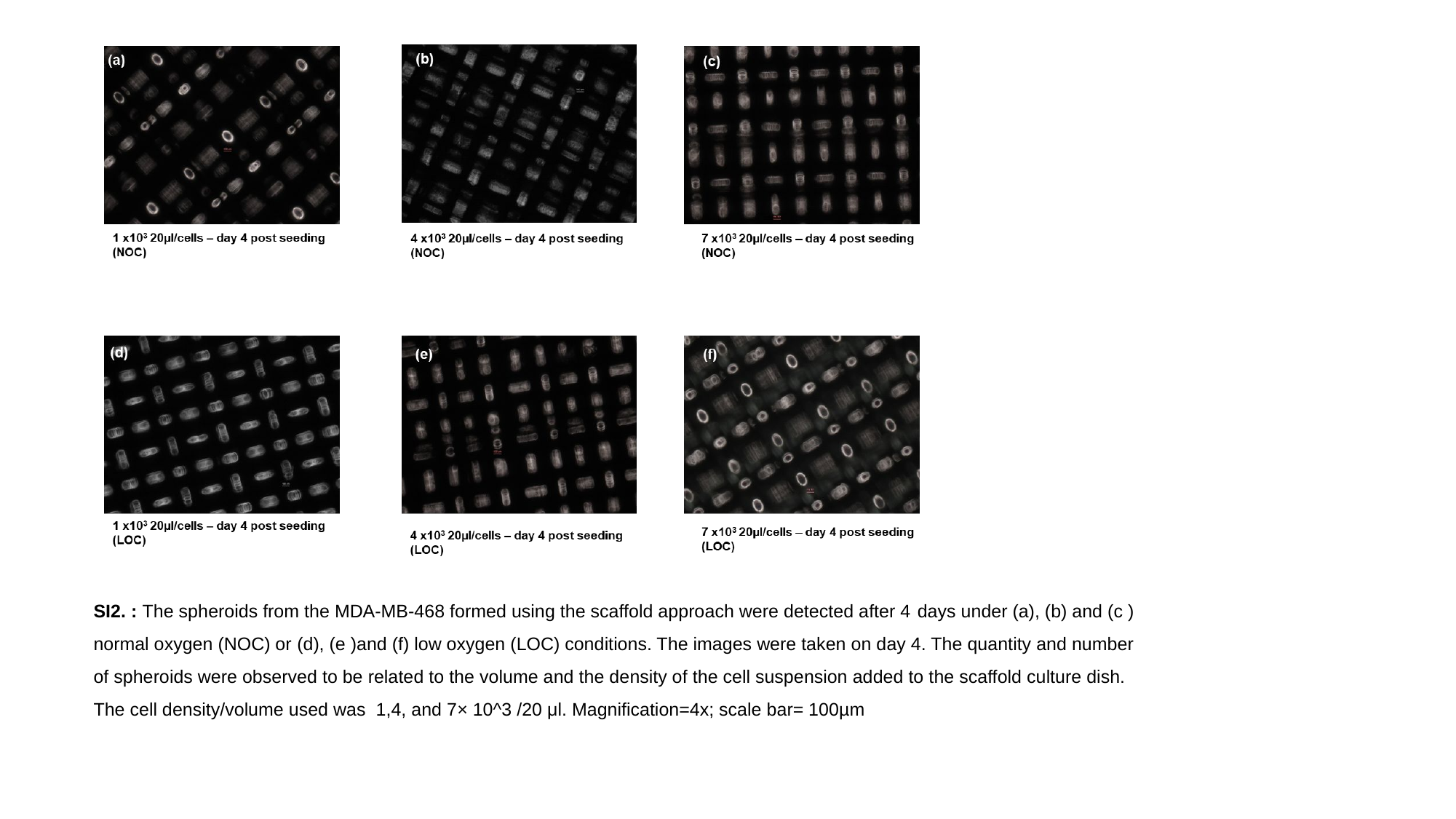

SI2. : The spheroids from the MDA-MB-468 formed using the scaffold approach were detected after 4 days under (a), (b) and (c ) normal oxygen (NOC) or (d), (e )and (f) low oxygen (LOC) conditions. The images were taken on day 4. The quantity and number of spheroids were observed to be related to the volume and the density of the cell suspension added to the scaffold culture dish. The cell density/volume used was 1,4, and 7× 10^3 /20 μl. Magnification=4x; scale bar= 100µm
