# Supplementary material for: Optimising the method to develop spheroids from MDA-MB-468 human triple negative breast cancer cells
Source: Mol Biol Rep. 2026 Jan 24;53(1):322. doi: 10.1007/s11033-026-11451-4 (PMC12831690; doi:10.1007/s11033-026-11451-4)
Supplement: Supplementary file 2 — Supplementary Material 2 [file 11033_2026_11451_MOESM2_ESM.pdf]

Additional details on methods

Image selection:

For all 3D conditions (ULA, agarose, rigid scaffold), quantitative morphometrics (equivalent diameter, area, perimeter, circularity, solidity) were extracted only from fully contained objects that met pre-specified quality criteria; edge-touching or truncated objects were excluded from size analyses.

Acquisition and calibration. Bright-field images were acquired under harmonised settings (identical objective/magnification, illumination/exposure/gain, pixel size, and display range). Spatial calibration was applied uniformly from the microscope scale bar, enabling size computation in micrometres.

inclusion/exclusion (applies to all methods).

Include only objects with a closed, continuous boundary fully contained within the frame (no edge contact), adequate focus for boundary detection (≥3 adjacent z-planes for scaffold), and meeting our a priori morphometric thresholds (circularity ≥ 0.85; solidity ≥ 0.9; minimum equivalent diameter ≥ 50 μm for size analyses).

Exclude edge-touching/truncated objects, overlapping/merging aggregates, tethered fragments, or out-of-focus candidates.

Scaffold-specific handling: On the rigid scaffold, imaging was performed in situ to avoid mechanical disturbance. Because pores/struts can create bright contours, a candidate object was accepted only if a cellular boundary could be traced independently of the strut edge and remained stable across z-planes. Representative figure panels show cropped views for readability; all measurements were taken from the corresponding full-field frames acquired at the same session.

Longitudinal panels and mobile aggregates. For the longitudinal examples (day 4, 5 and 6), the same well/field was followed at each time point. Apparent xy shifts of circled objects

reflect free movement in U-bottom wells and gentle medium exchange, not a change in the object images. Only objects that remained fully contained at a given time point were measured at that time; if an object became edge-touching later, it was excluded for that time point (and the reduced number of replicates is reflected in the legend/Methods) and was not included in the presented images; however, it will be reported

Fluorescence images: Fluorescent panels were used qualitatively (e.g., Hoechst nuclear distribution) and were not used for size quantitation. All quantitative size metrics derive from the harmonised bright-field workflow described above.
