# Supplementary material for: Optimising the method to develop spheroids from MDA-MB-468 human triple negative breast cancer cells
Source: Mol Biol Rep. 2026 Jan 24;53(1):322. doi: 10.1007/s11033-026-11451-4 (PMC12831690; doi:10.1007/s11033-026-11451-4)
Supplement: Supplementary file 3 — Supplementary Material 3 [file 11033_2026_11451_MOESM3_ESM.pdf]

SI Table1: Primers used for the quantitative polymerase chain reaction

Gene Symbol	Gene Name	Primer Sequence (5' - 3')	
HIF1A	*Hypoxia-inducible factor-1-alpha*	**F:**	CCT CTG TGA TGA GGC TTA CCA TC
		**R:**	CAT CTG TGC TTT CAT GTC ATC TTC
NES	*Nestin*	**F:**	CTT CCC TCC GCA TCC CGT CA
		**R:**	AAA GCC AGC ATG TCA CCC TC
SNAI1	*Snail family transcriptional repressor-1*	**F:**	CGG TTC CGA TGC CCT GAG GCT C
		**R:**	CGT CAC ACT TCA TGA TGG AAT TG
TWIST1	*Twist basic helix-loop-helix transcription*	**F:**	ACC ATC CTC ACA CCT CTG CAT
		**R:**	TTC CTT TCA GTG GCT GAT TGG
VEGFA	*Vascular endothelial growth factor A*	**F:**	AGG GCA GAA TCA TCA CGA AG
		**R:**	CAC ACA GGA TGG CTT GAA G
CD44	*Cluster of differentiation 44*	**F:**	CGC CAA ACA CCC AAA GAA
		**R:**	GTG TTG TCC TTC CTT GCA TT
GAPDH	*Housekeeping gene*	**F:**	ACC ACA GTC CAT GCC ATC AC
		**R:**	TCC ACC ACC CTG TTG CTG TA

F: Forward; **R:** Reverse;

SI Table 1: Comparison of Spheroid Culture Techniques in MDA-MB-468 TNBC Cells under Varying Oxygen Conditions in this study

Parameter	Hanging Drop (HD)	Liquid Overlay (ULA/Agarose) *	Scaffold-Based
Spheroid Formation Time	Moderate (24–36 h)	Dependent on HD pre-formation	Fast (24–48 h)
Structural Integrity	Moderate under (NOC)	High (especially ULA under NOC)	Variable (less compact under LOC)
Morphometric Uniformity (Sphericity)	Variable	High under NOC, moderate under LOC	Moderate; perimeter fluctuation observed
Ease of Handling & Imaging	Moderate; prone to disruption	High (ULA) / Moderate (Agarose)	Low (scaffold material obscures visualisation)
Gene Expression Consistency	Moderate	High (notably *CD44*, *TWIST1, VEGFA* under NOC)	less responsive under LOC
Hypoxia Simulation (*HIF1A*, *VEGFA*)	Poor under LOC	NOC mimics pseudo-hypoxia response	Inconsistent, sometimes inverse expression
Reproducibility	Moderate	High	Moderate

LOC: low oxygen condition; **HD:** hanging drop; **NOC:** normal oxygen condition; **ULA:** ultra-low attachment; *;Rat tail collagen I added to the complete media
