# Supplementary material for: Optimising the method to develop spheroids from MDA-MB-468 human triple negative breast cancer cells
Source: Mol Biol Rep. 2026 Jan 24;53(1):322. doi: 10.1007/s11033-026-11451-4 (PMC12831690; doi:10.1007/s11033-026-11451-4)
Supplement: Supplementary file 4 — Supplementary Material 4 [file 11033_2026_11451_MOESM4_ESM.pdf]

(a)

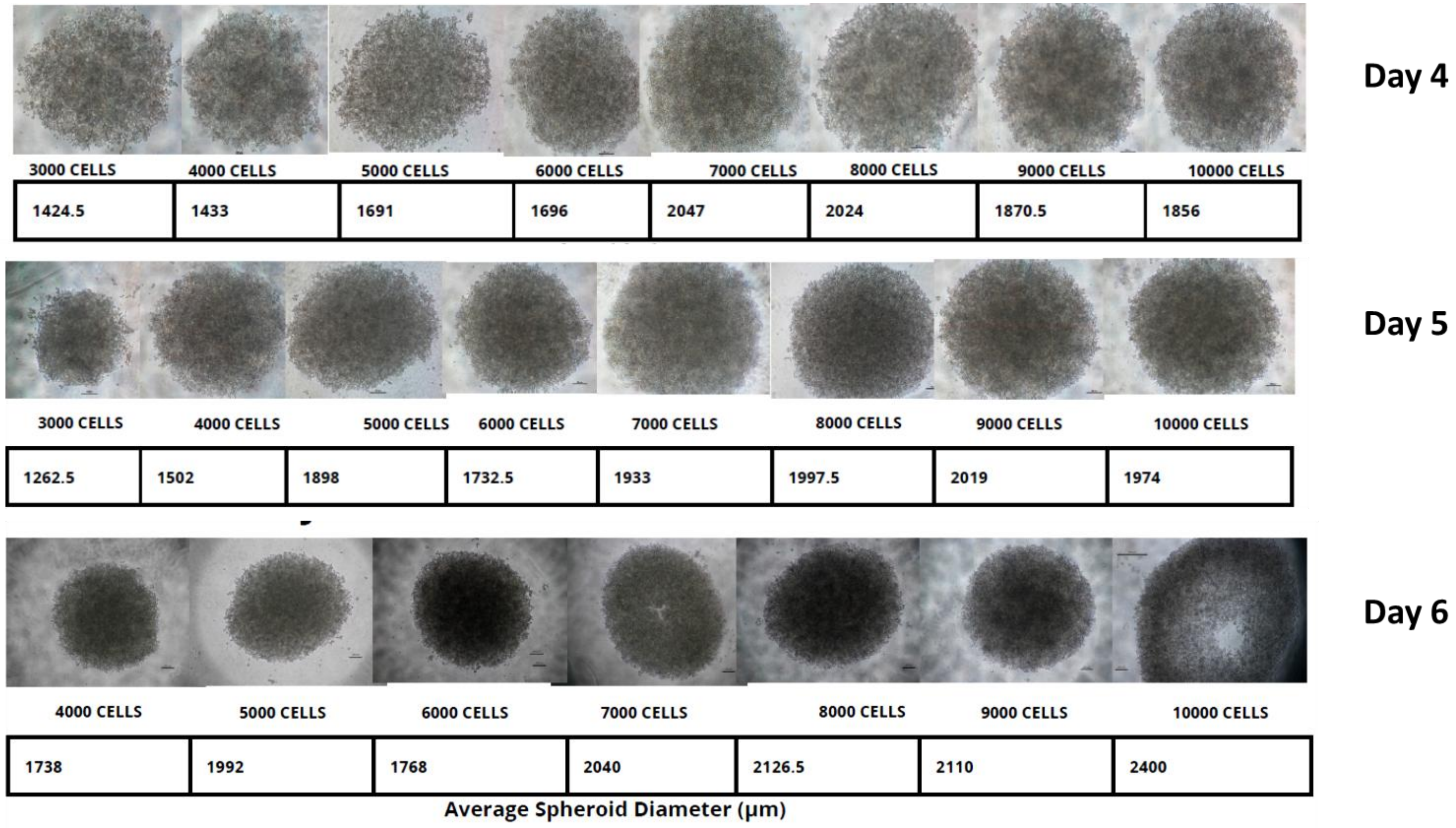

SI1. a: Three-dimensional (3D) culture developed from MDA-MB-468 TNBC cells [(1-10) × 10^3 cells/20 μL] in complete medium. The average change in diameters (depicted in the boxes below the image in microns)of the 3D culture was recorded on days 4, 5, and 6 post-seeding under normal oxygen conditions (NOC).The best cellular aggregate was observed when 4–6× 10^3 /20μL.

Magnifications=4x; scale bar=100μm

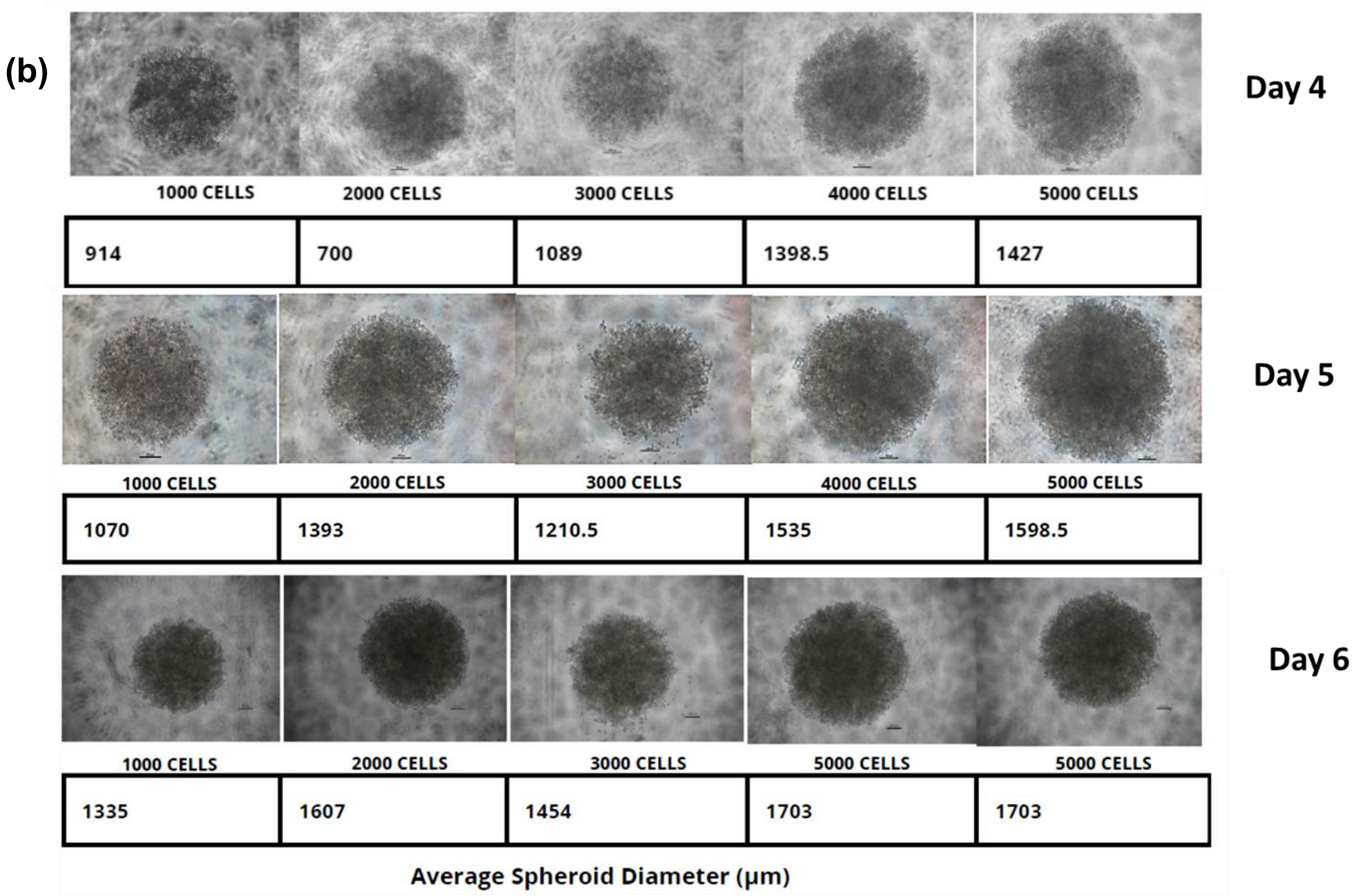

SI1. b: Three-dimensional (3D) culture developed from MDA-MB-468 TNBC cells [(1-5) × 10^3 cells/20 μL] in complete medium. The average change in diameters (depicted in the boxes below the image in microns) of the 3D culture was recorded on days 4, 5, and 6 post-seeding under low oxygen conditions (LOC). The best cellular aggregate was observed when 3–5× 10^3 /20μL**. Magnifications=4x; scale bar=100μm**

(a)

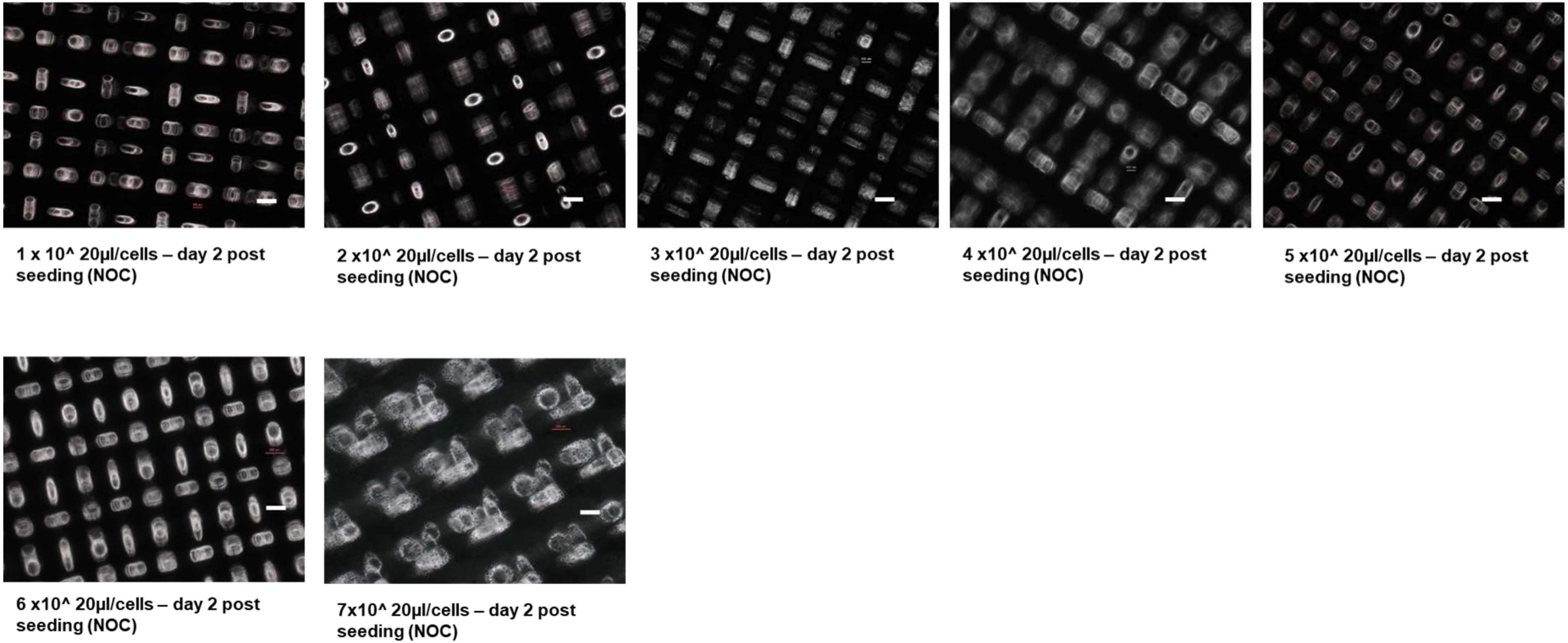

SI2. a: The spheroids from the MDA-MB-468 formed using the scaffold approach were detected starting from day 2 post-seeding under normal oxygen conditions (NOC). Images were taken on day 2. The quantity and number of spheroids were observed to be related to the volume and the density of the cell suspension added to the scaffold culture dish. The cell density/volume used was 1-7 × 10^3 /20 µL. Magnification=4x; scale bar in white = 200µm

(b)

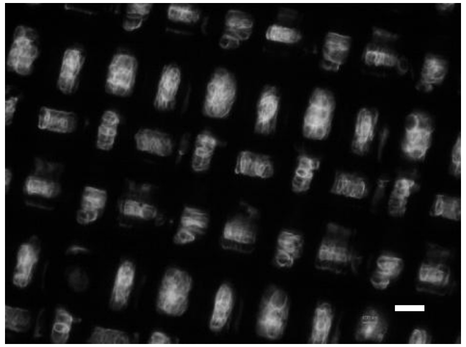

1 x 10^ 20µl/cells – day 2 post seeding (LOC)

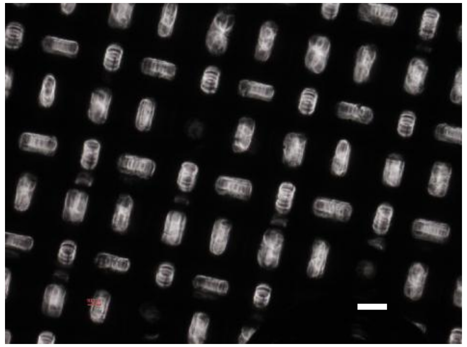

2 x10^ 20µl/cells – day 2 post seeding (LOC)

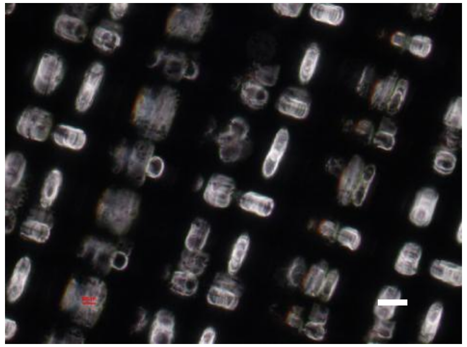

3 x10^ 20µl/cells – day 2 post seeding (LOC)

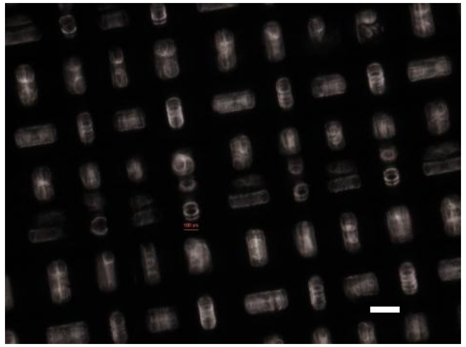

4 x10^ 20µl/cells – day 2 post seeding (LOC)

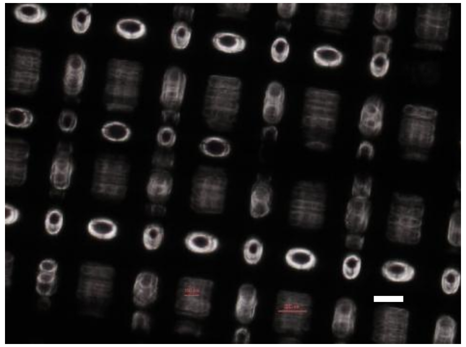

5 x10^ 20µl/cells – day 2 post seeding (LOC)

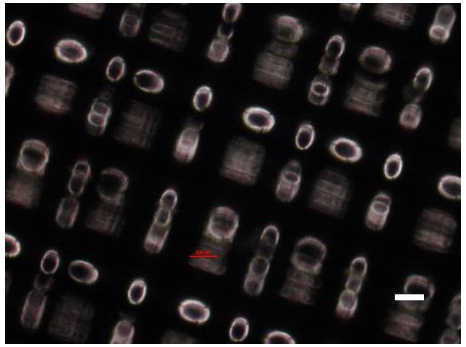

6 x10^ 20µl/cells – day 2 post seeding (LOC)

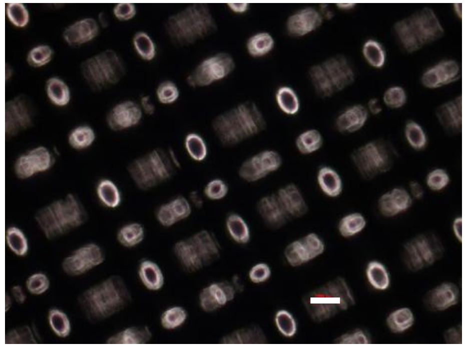

7x10^ 20µl/cells – day 2 post seeding (LOC)

SI2. b: The spheroids from the MDA-MB-468 formed using the scaffold approach were detected starting from day 2 post-seeding under low oxygen conditions (LOC). Images were taken on day 2. The quantity and number of spheroids were observed to be related to the volume and the density of the cell suspension added to the scaffold culture dish. The cell density/volume used was 1-7 × 10^3 /20 µL. Magnification=4x; scale bar in white = 200µm

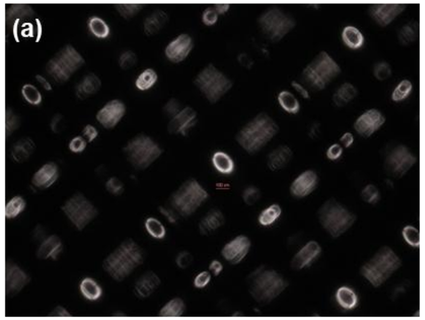

1 x10^3 20µl/cells – day 4 post seeding (NOC)

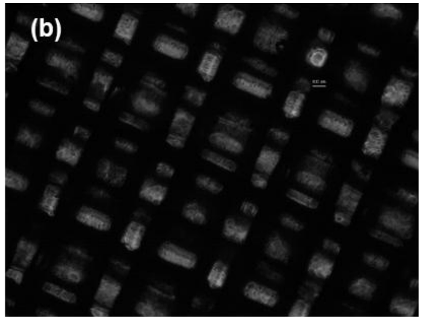

4 x10^3 20µl/cells – day 4 post seeding (NOC)

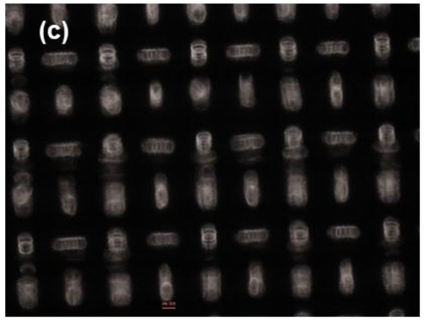

7 x10^3 20µl/cells – day 4 post seeding (NOC)

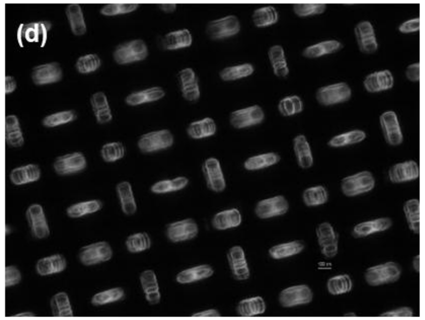

1 x10^3 20µl/cells – day 4 post seeding (LOC)

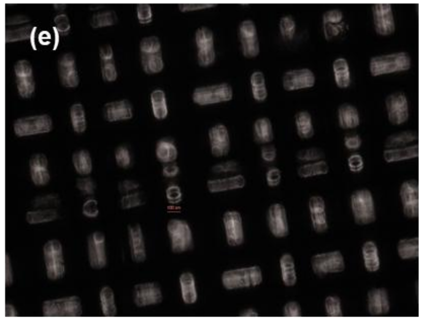

4 x10^3 20µl/cells – day 4 post seeding (LOC)

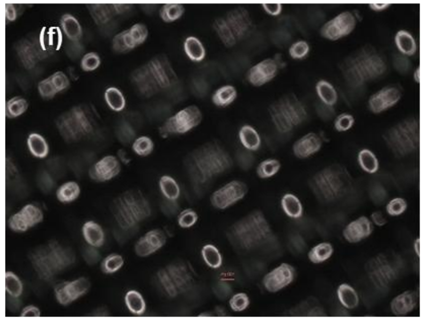

7 x10^3 20µl/cells – day 4 post seeding (LOC)

SI3. : The spheroids from the MDA-MB-468 formed using the scaffold approach were detected after 4 days under (a), (b) and (c) normal oxygen (NOC) or (d), (e)and (f) low oxygen (LOC) conditions. The images were taken on day 4. The quantity and number of spheroids were observed to be related to the volume and the density of the cell suspension added to the scaffold culture dish. The cell density/volume used was 1,4, and 7× 10^3 /20 µl. Magnification=4x; scale bar= 100µm

[A] [B] [C]

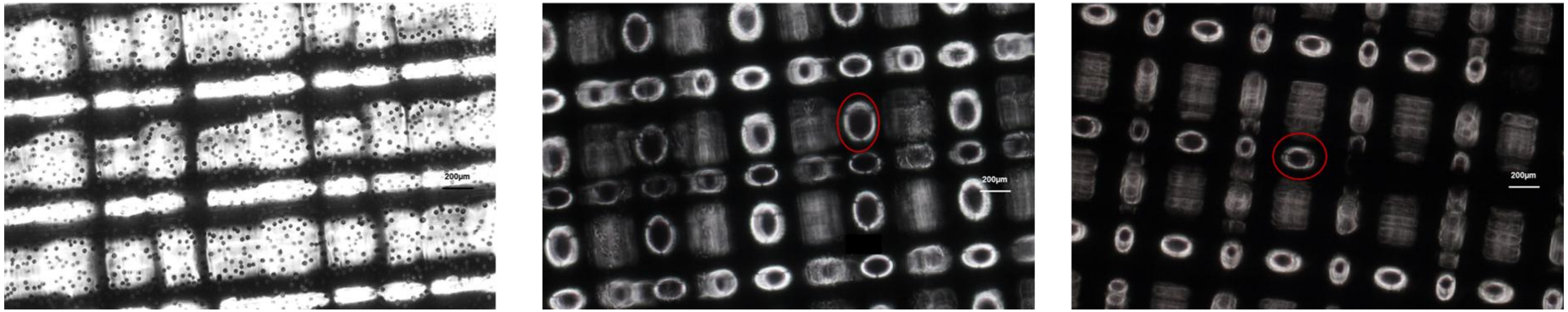

SI4: Formation of MDA-MB-468 spheroids using the 3D scaffold culture [A] Day 0 post-seeding image showing single MDA-MB-468 cells distributed on the scaffold surface. [B] normal (NOC) and [C] low oxygen conditions showing the appearance of early 3D compact aggregate formation (red circles). The cell density/volume used was 5×10^3 /20 µL / 1.5 mL. [B] and [C] captured on day 2 post-seeding..
Magnification 4x; Scale bar = 200µm

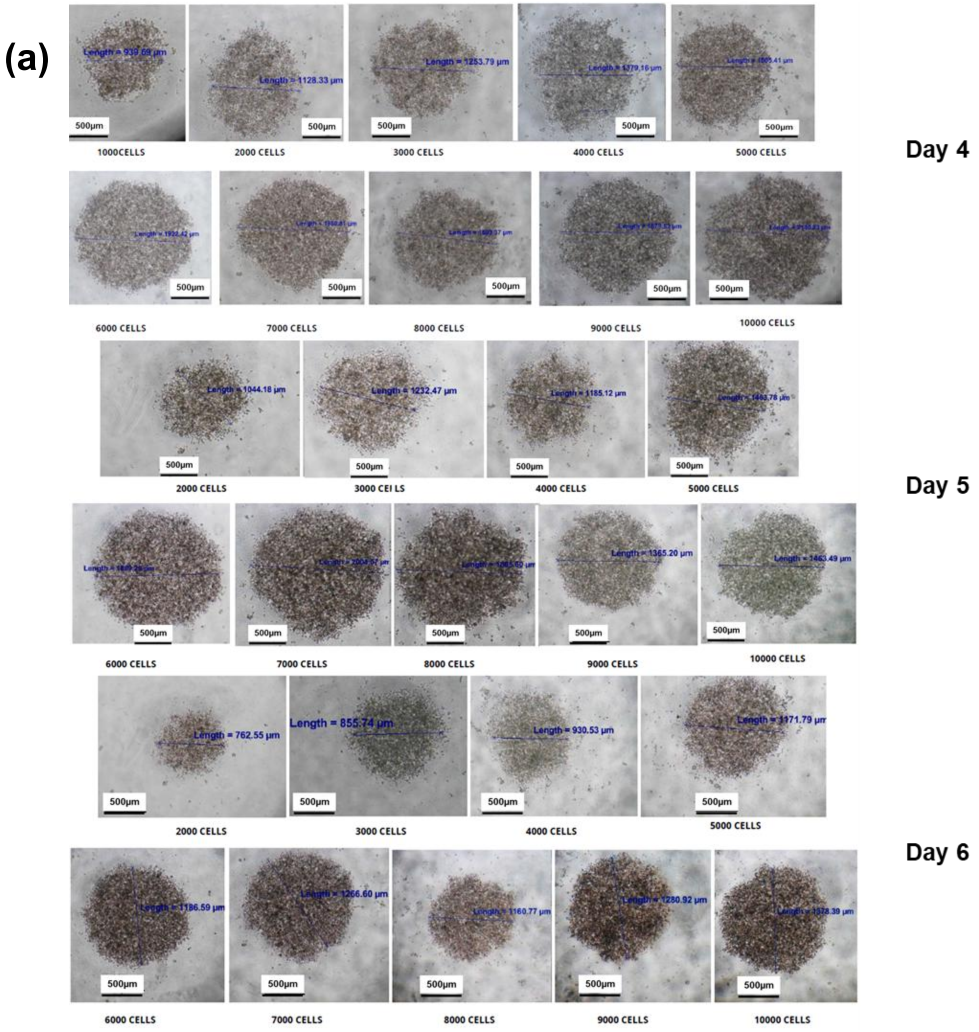

SI5 a: Three-dimensional (3D) culture developed from MDA-MB-231 TNBC cells [(1-10) × 10^3 cells/20 µL] in complete medium. The average change in diameters (depicted on the images in microns)of the 3D culture was recorded on days 4, 5, and 6 post-seeding under normal oxygen conditions (NOC).The best cellular aggregate was observed when 4–7× 10^3 /20µL.

Magnifications = 4x; scale bar = 500µm

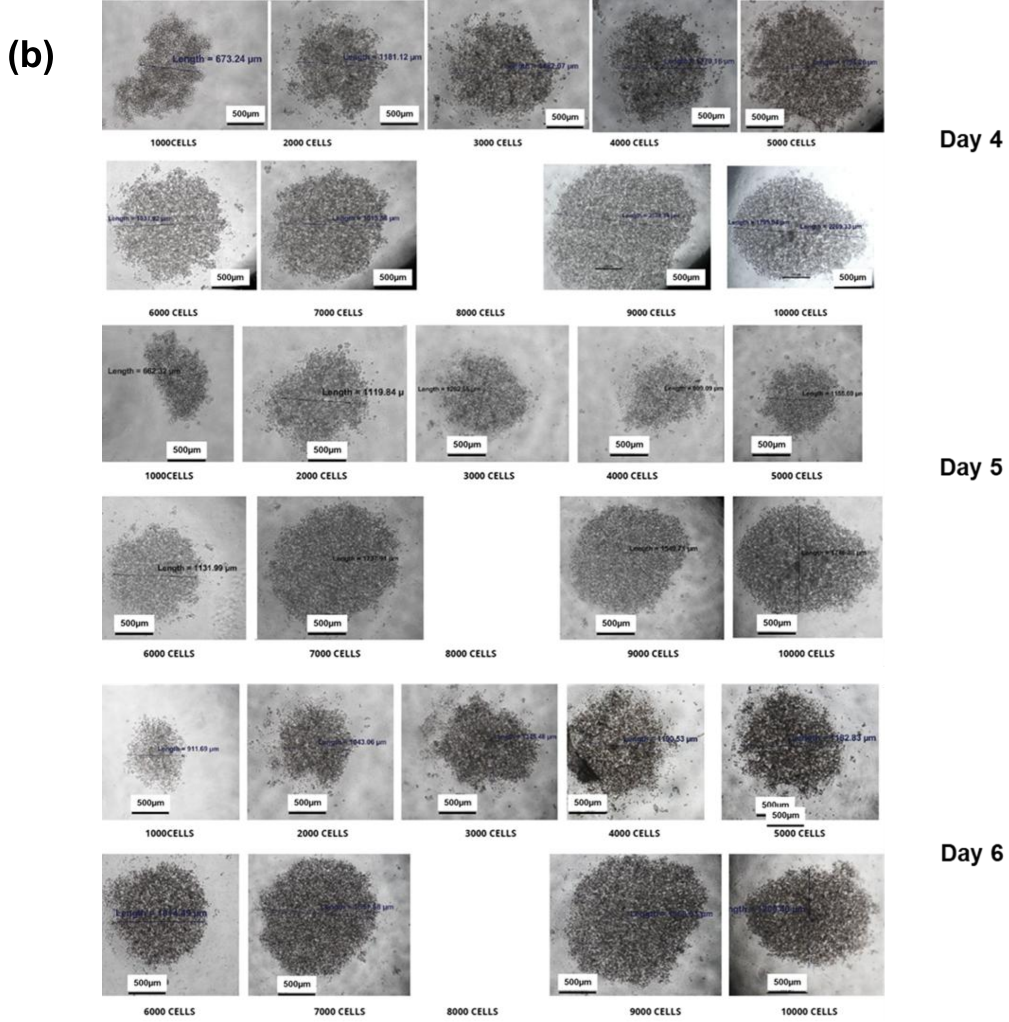

SI5 b: Three-dimensional (3D) culture developed from MDA-MB-231 TNBC cells [(1-10) × 10^3 cells/20 µL] in complete medium. The average change in diameters (depicted on the images in microns) of the 3D culture was recorded on days 4, 5, and 6 post-seeding under low oxygen conditions (LOC). HD yielded the most uniform, compact spheroids at 4–7 × 10^3 cells/20 µL. No 3D aggregates were formed at 8x 10^3 cells/20 µL cell density. **Magnifications= 4x; scale bar = 500µm**

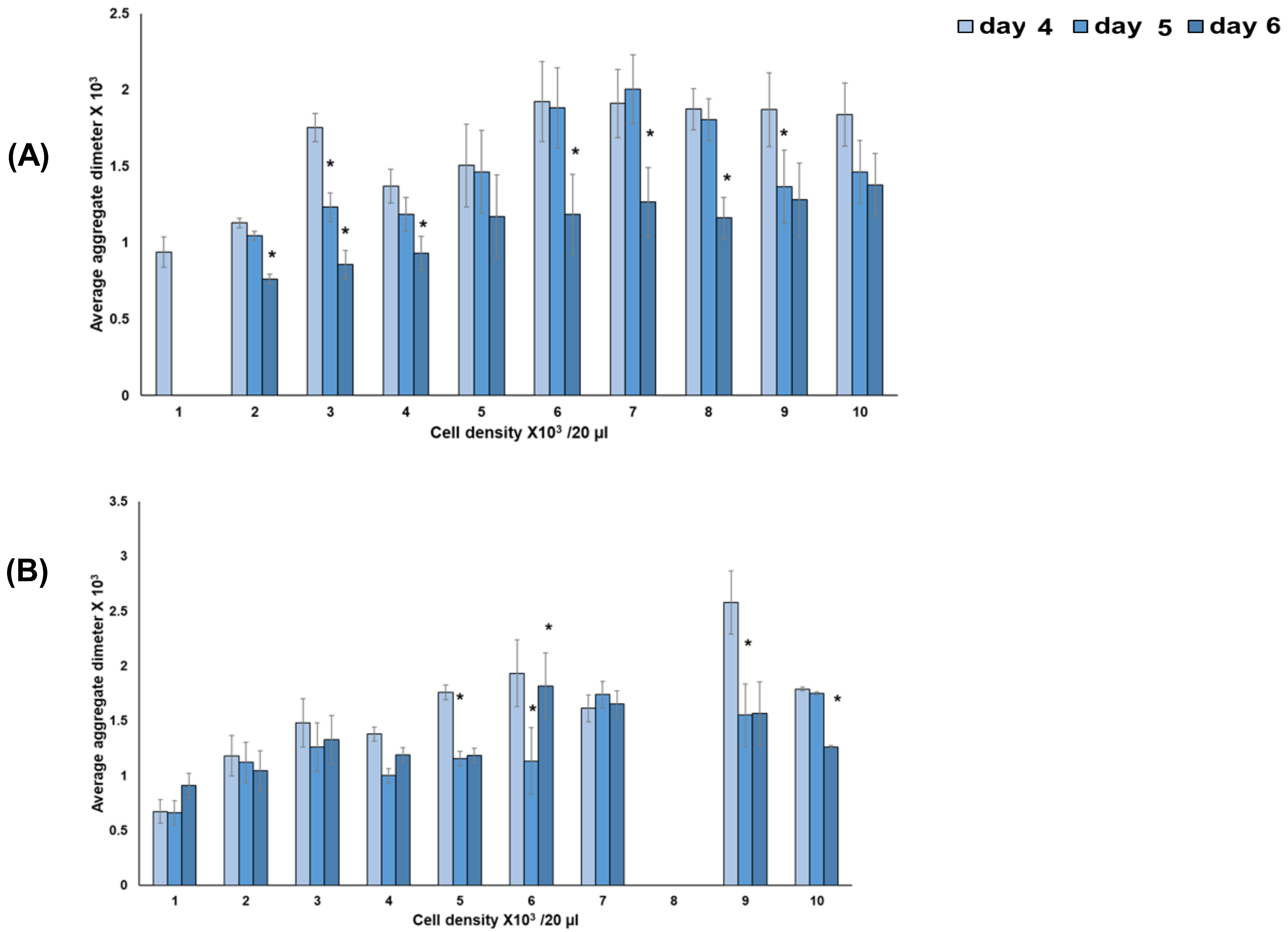

SI6:3D cultures formation by MDA-MB-231 TNBC cells across seeding densities and oxygen conditions. Cells were seeded at (1–10) × 10^3 cells/20 µL in complete medium and cultured at 37 °C, 5% CO_2 under (**A**) normal oxygen (NOC) or (**B**) low oxygen (LOC). Mean percentage change in spheroid diameter was recorded on days 4, 5, and 6. Under NOC, HD yielded the most uniform, compact spheroids at 4–7 × 10^3 cells/20 µL. Under LOC no 3d aggregates were formed at 8x 10^3 cells/20 µL. Data are shown as mean ± SD; two-way ANOVA, $p < 0.05$

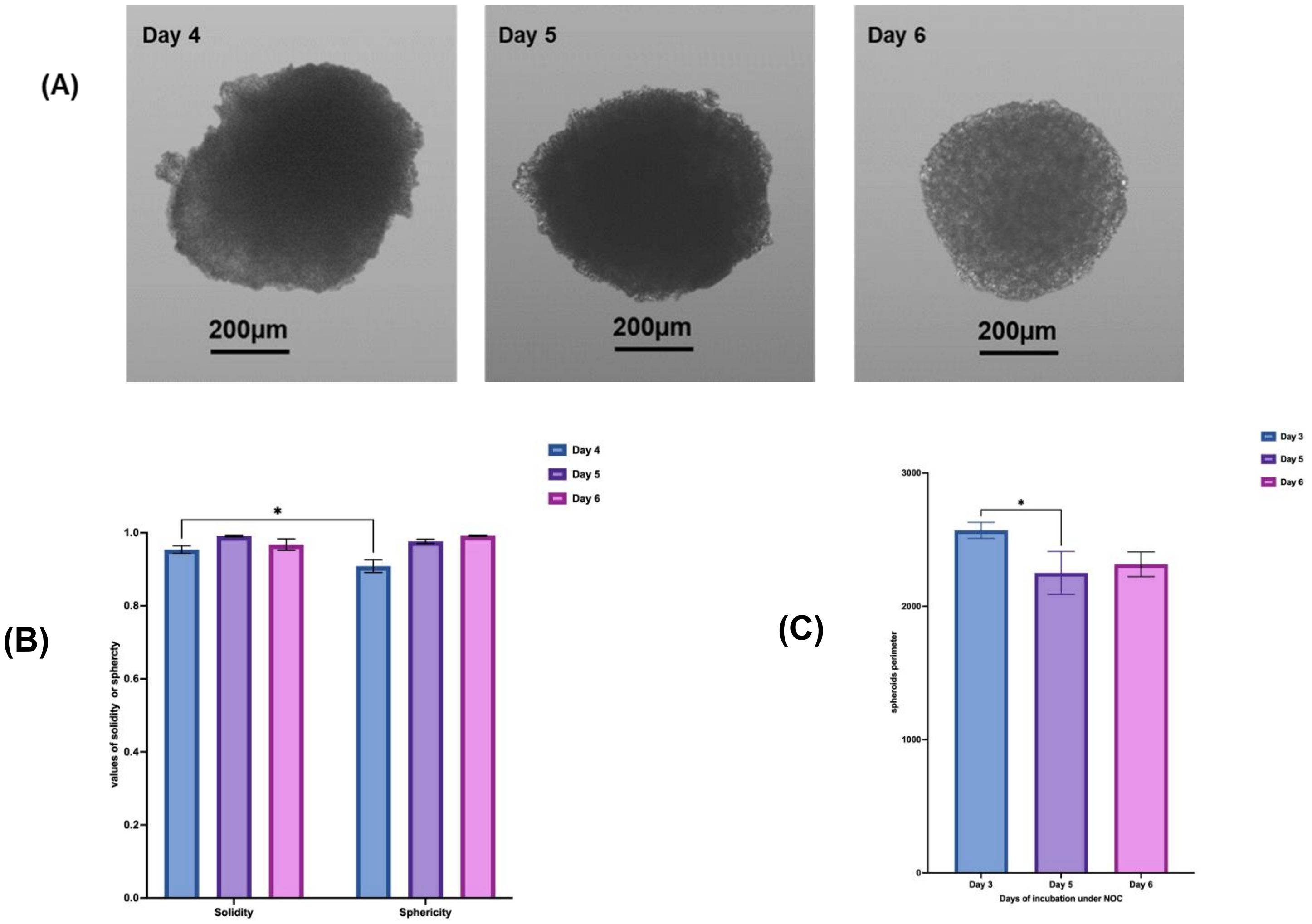

SI7:3D culture formation of MDA-MB-231 TNBC cells was assessed using ultra-low attachment (ULA) plates under normal (NOC). **[B,C]**. Morphometric parameters, including solidity, sphericity, and perimeter, were quantified using AnaSP image analysis software. Images are representative of a single well that was followed longitudinally across the captured time period. Red arrows indicate cellular debris. Data are expressed as the mean ± standard error of the mean (SEM) from three independent biological replicates. Statistical significance was determined as $p < 0.05$ (*) using one-way ANOVA. Spheroid images were captured using an inverted light microscope (Nikon Eclipse TE2000-U) at 4× magnification. **NOC: normal oxygen condition; scale bar=200 micron**

SI Table1:Primers used for the quantitative polymerase chain reaction

Gene Symbol	Gene Name		Primer Sequence (5’ - 3’)
HIF1A	*Hypoxia-inducible factor-1-alpha*	**F:**	CCT CTG TGA TGA GGC TTA CCA TC
		**R:**	CAT CTG TGC TTT CAT GTC ATC TTC
NES	*Nestin*	**F:**	CTT CCC TCC GCA TCC CGT CA
		**R:**	AAA GCC AGC ATG TCA CCC TC
SNAI1	*Snail family transcriptional repressor-1*	**F:**	CGG TTC CGA TGC CCT GAG GCT C
		**R:**	CGT CAC ACT TCA TGA TGG AAT TG
TWIST1	*Twist basic helix-loop-helix transcription*	**F:**	ACC ATC CTC ACA CCT CTG CAT
		**R:**	TTC CTT TCA GTG GCT GAT TGG
VEGFA	*Vascular endothelial growth factor A*	**F:**	AGG GCA GAA TCA TCA CGA AG
		**R:**	CAC ACA GGA TGG CTT GAA G
CD44	*Cluster of differentiation 44*	**F:**	CGC CAA ACA CCC AAA GAA
		**R:**	GTG TTG TCC TTC CTT GCA TT
GAPDH	*Housekeeping gene*	**F:**	ACC ACA GTC CAT GCC ATC AC
		**R:**	TCC ACC ACC CTG TTG CTG TA

F: Forward; **R:** Reverse;

SI Table 2: Comparison of Spheroid Culture Techniques in MDA-MB-468 TNBC Cells under Varying Oxygen Conditions in this study

Parameter	Hanging Drop (HD)	Liquid Overlay (ULA/Agarose)*	Scaffold-Based
Spheroid Formation Time	Moderate (24–36 h)	Dependent on HD pre-formation	Fast (24–48 h)
Structural Integrity	Moderate under (NOC)	High (especially ULA under NOC)	Variable (less compact under LOC)
Morphometric Uniformity (Sphericity)	Variable	High under NOC, moderate under LOC	Moderate; perimeter fluctuation observed
Ease of Handling & Imaging	Moderate; prone to disruption	High (ULA) / Moderate (Agarose)	Low (scaffold material obscures visualisation)
Gene Expression Consistency	Moderate	High (notably *CD44*, *TWIST1, VEGFA* under NOC)	less responsive under LOC
Hypoxia Simulation (*HIF1A*, *VEGFA*)	Poor under LOC	NOC mimics pseudo-hypoxia response	Inconsistent, sometimes inverse expression
Reproducibility	Moderate	High	Moderate

LOC: low oxygen condition; **HD:** hanging drop; **NOC:** normal oxygen condition; **ULA:** ultra-low attachment; *;Rat tail collagen I added to the complete media
